# Delivery of intraflagellar transport proteins to the ciliary base and assembly into trains

**DOI:** 10.1126/sciadv.adr1716

**Published:** 2025-04-04

**Authors:** Aniruddha Mitra, Evangelos Gioukakis, Wouter Mul, Erwin J. G. Peterman

**Affiliations:** Department of Physics and Astronomy and LaserLaB, Vrije Universiteit Amsterdam, Amsterdam, Netherlands.

## Abstract

Anterograde intraflagellar transport (IFT) trains, composed of IFT-B, IFT-A, and BBSome subcomplexes, are responsible for transporting ciliary proteins into the cilium. How IFT subcomplexes reach the ciliary base and assemble into IFT trains is poorly understood. Here, we perform quantitative single-molecule imaging in *Caenorhabditis elegans* chemosensory cilia to uncover how IFT subcomplexes arrive at the base, organize in IFT trains, and enter the cilium. We find that BBSomes reach the base via diffusion where they either associate with assembling IFT trains or with the membrane surrounding the base. In contrast, IFT-B and IFT-A reach the base via directed transport most likely on vesicles that stop at distinct locations near the base. Individual subcomplexes detach from the vesicles into a diffusive pool and associate to assembling trains. Our results show that IFT-B is first incorporated into IFT trains, followed by IFT-A, and finally BBSomes, indicating that the assembly of IFT trains is a highly regulated, step-wise process.

## Teaser

IFT subcomplexes use different means to reach ciliary base and incorporate into intraflagellar transport trains that enter cilia.

## INTRODUCTION

**Fig. 1.**
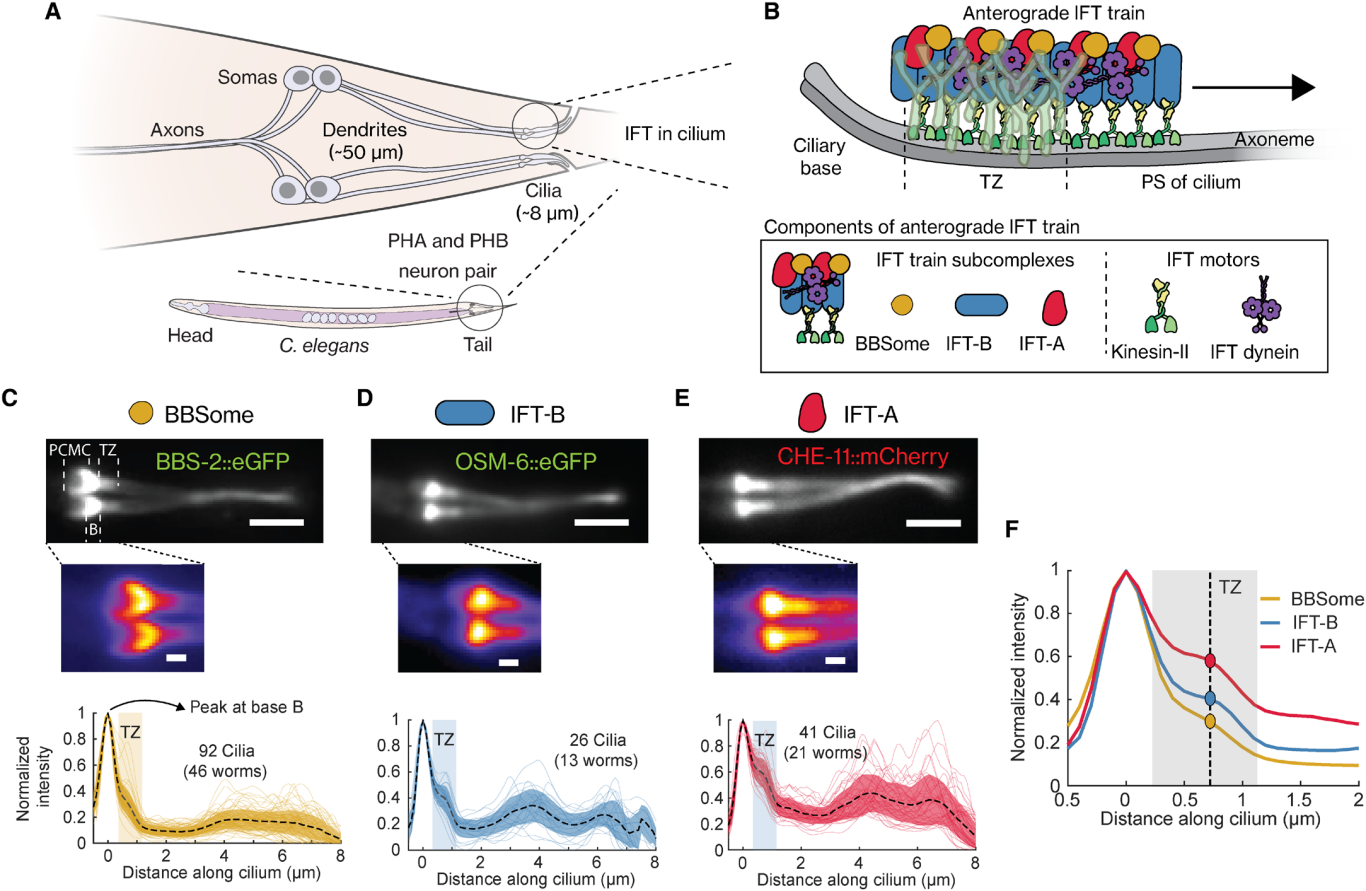
BBSome, IFT-B, and IFT-A show distinct localization patterns near the ciliary base of PHA/PHB cilia. (**A**) Schematic diagram of the two pairs of phasmid neurons (PHA/PHB) located at the tail of *C. elegans*. These neurons have ~50-μm-long dendrites that connect the somas to the cilia (~8 μm long), with the ciliary tip protruding out of the worm body. (**B**) Top, illustration of IFT of an anterograde IFT train crossing the TZ [indicated by the Y-shaped linkers (cyan)] to enter the proximal segment (PS) of the cilium. Bottom, components of an anterograde IFT train. (**C** to **E**) Representative time-averaged fluorescence images (top, corresponding movies in movie S1; insets below show zoom-in of the ciliary base), and the average fluorescence-intensity profiles (bottom, plotting means ± SD) obtained from multiple cilia, for (C) BBSome (eGFP::BBS-2, an IFT-train subcomplex BBSome subunit; 92 cilia from 46 worms), (D) IFT-B (OSM-6::eGFP, an IFT-train subcomplex B subunit; 26 cilia from 13 worms), and (E) IFT-A (CHE-11::mCherry, an IFT-train subcomplex A subunit; 41 cilia from 21 worms). All fluorescence-intensity profiles are normalized to the peak at the ciliary base, and the profiles from individual cilia are also plotted in the background. Scale bar, 2 μm (inset, 0.5 μm). PCMC, periciliary membrane compartment; B, ciliary base; TZ, transition zone. (**F**) The average fluorescence-intensity profile of BBSome, IFT-B, and IFT-A, with the shaded area roughly indicating the location of the TZ and the dotted line indicating the center of the TZ. The relative intensity at the TZ with respect to the peak at the ciliary base is different for the IFT-train subcomplexes.

Primary or sensory cilia are essential, “antenna-like” organelles protruding from most eukaryotic cells to sense the external environment and act as signal transducers (*1*). Ciliary structure and function are maintained by a highly regulated transport process, referred to as intraflagellar transport (IFT), where IFT trains, driven by kinesin-2 and IFT-dynein motors, ferry ciliary proteins from base to tip and back again along a microtubule-based axoneme (*2*, *3*). At the base, a ciliary gate, composed of transition fibers and the transition zone (TZ), restricts the diffusive entry and exit of both membrane-bound and soluble proteins (*4*, *5*), with only small cytosolic proteins (<30 kDa) able to freely diffuse across the barrier (*6*, *7*). To enter, several ciliary proteins have been shown to associate with anterograde IFT trains, assembled at the ciliary base, to be transported through the ciliary gate (Fig. 1B). Anterograde IFT trains are ordered polymeric structures (>80 MDa) with periodic repeats of IFT-B subcomplexes (each IFT-B complex consisting of 16 proteins) at their core (*8*). To this core, IFT-A subcomplexes (nine proteins) attach, positioned away from the axoneme facing the ciliary membrane, with a periodicity suggesting an IFT-B/IFT-A stoichiometry of 2:1 (*9*). How the third subcomplex, the BBSome (complex of eight BBS proteins) associates with anterograde IFT trains remains unknown. In recent years, fluorescent recovery after photobleaching (FRAP) (*10*, *11*) and structural (*12*) studies in *Chlamydomonas* and *Xenopus* embryos, as well as a single-molecule fluorescence study in *Caenorhabditis elegans* (*13*), have revealed a structural and dynamic picture of how anterograde IFT trains assemble at the base. It appears that IFT trains tether from one end to the TZ while being assembled, with IFT-B forming a scaffold to which IFT-A and IFT-dynein attach subsequently from the dendritic side (*12*). Kinesin-2 motors only associate with trains just before the trains’ departure, while the cargo tubulin attaches to fully assembled and moving trains (*11*, *13*). So far, it has not been investigated when and where the BBSome assembles to IFT trains. An additional open question is how IFT components are transported from their sites of synthesis in the soma (via the dendrite in *C. elegans* chemosensory cilia) to the ciliary base and are organized for assembly into trains. In recent years, IFT proteins have been linked to an extraciliary role in vesicular trafficking (*14*, *15*). Several IFT-B and IFT-A proteins appear to share a common ancestry with classical vesicular coat proteins (COPs) (*16*, *17*), and electron and light-microscopy studies have revealed that some of these proteins associate with periciliary vesicles (*18*–*21*). Because of this, it has been proposed that some IFT components might reach the ciliary base via active microtubule-based transport. A recent FRAP study in *Xenopus*, however, has indicated that disruption of cytoplasmic microtubules does not affect the recruitment of IFT components at the ciliary base (*10*). This led the authors to propose that the IFT components form a diffusive pool near the base, with proteins assembling onto trains via a diffusion-to-capture mechanism (*22*). While recent studies provide important insights in the ensemble dynamics of IFT components outside cilia, a clear picture at the single-molecule level is still lacking.

In this study, we perform single-particle imaging to visualize the dynamics of individual fluorescently labeled IFT components in the ciliated chemosensory neurons of *C. elegans*. We focus on the PHA/PHB neuron pairs located at the tail of the worms. In these neurons, the cilium is not directly connected to the soma but via the ~40- to 50-μm-long dendrite (Fig. 1A). Single-particle tracking and analysis reveal how IFT subcomplexes (BBSome, IFT-B, and IFT-A) and IFT motors (kinesin-II and IFT-dynein) are transported through the dendrite, from their sites of synthesis in the soma to the periciliary membrane compartment (PCMC; located at the transition between the dendrite and cilium). Furthermore, data and analysis reveal a comprehensive picture of the sorting dynamics of individual BBSome, IFT-B, and IFT-A subcomplexes in the PCMC as well as their interaction dynamics with anterograde IFT trains assembling at the ciliary base.

## RESULTS

### IFT-train subcomplexes have different localization patterns at ciliary base

To study IFT-train assembly in the PCMC and at the ciliary base, we imaged the ensemble dynamics of BBSome, IFT-A, and IFT-B subcomplexes in the chemosensory PHA/PHB neurons of live *C. elegans*. We used BBS-2::eGFP (enhanced green fluorescent protein) as a marker for the BBSome, OSM-6::eGFP (IFT52 in other organisms) as a marker for IFT-B, and CHE-11::eGFP for IFT-A (IFT140 in other organisms; see table S1 for a complete list of the strains used in this study). In the following, we will assume that these three proteins are always present in BBSome, IFT-B, and IFT-A subcomplexes, respectively, a point that will be discussed in detail later. Our ensemble fluorescence image sequences showed similar dynamics as observed before for these proteins (movie S1) (*23*, *24*). From these sequences, we obtained time-averaged fluorescence images and intensity profiles showing the ciliary distributions of BBSome (Fig. 1C), IFT-B (Fig. 1D), and IFT-A (Fig. 1E) subcomplexes. As shown before (*23*–*25*), the intensity distributions of these subcomplexes are roughly similar, all peaking at the ciliary base, with a shoulder in the TZ, decreasing in the proximal segment, and increasing again in the distal segment, where the two cilia of a PHA/PHB pair overlap. On the dendritic side of the TZ, however, IFT-A and IFT-B appeared to be more restricted to locations close to the ciliary base, while BBSome locations appeared to be extended further toward the PCMC (Fig. 1, C to E, inset). Furthermore, BBSome and IFT-B subcomplexes appeared to be more specifically enriched at the ciliary base than IFT-A (Fig. 1F), which shows a substantially larger shoulder in the TZ. These subtle differences in distributions of the BBSome, IFT-B, and IFT-A subcomplexes near the ciliary base suggest that these complexes are sorted differently before entry into the cilium.

### BBSomes diffuse to the ciliary base, while IFT subcomplexes are actively transported

**Fig. 2.**
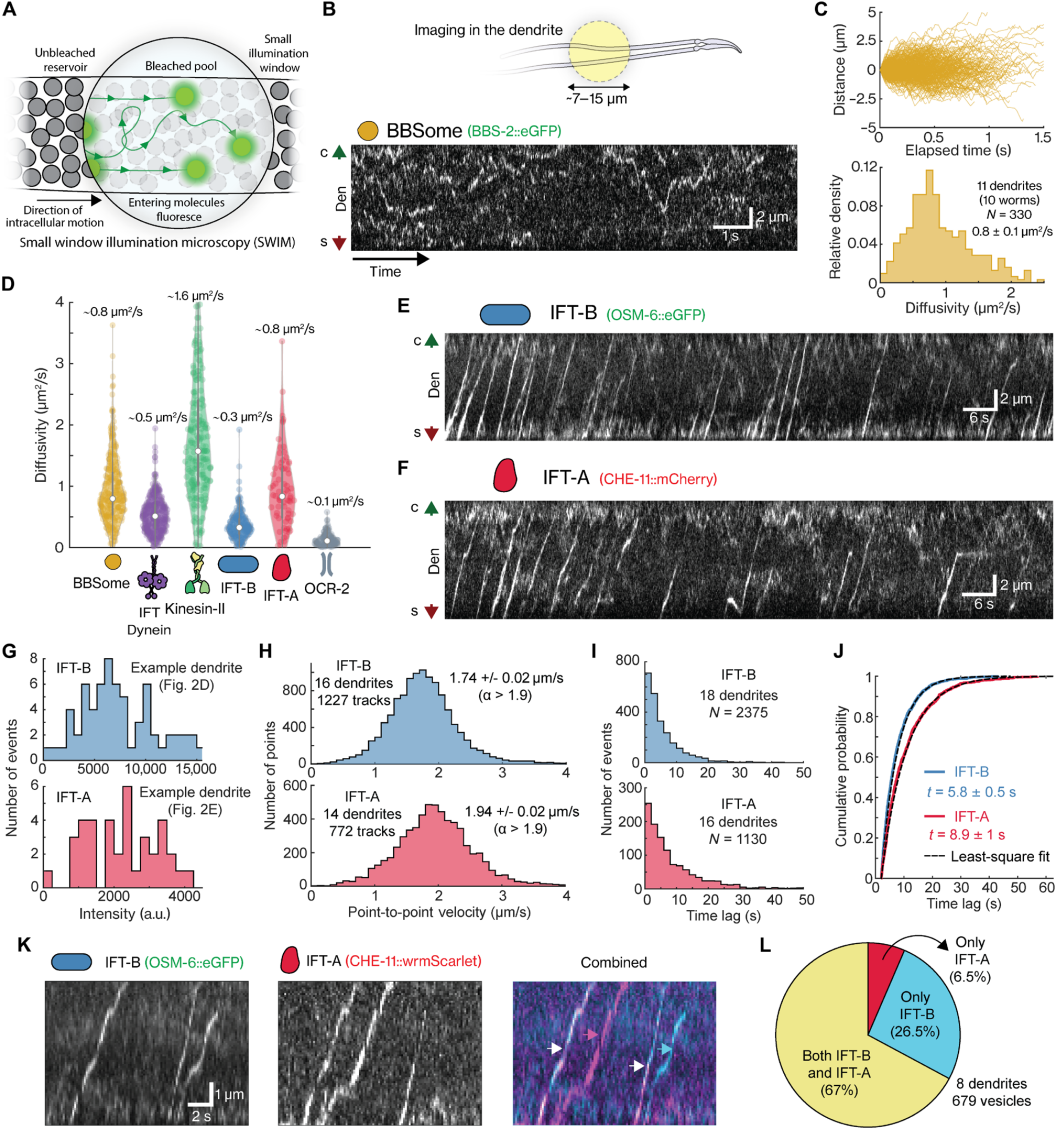
BBSome and IFT dynamics in the dendrite. (**A**) Illustration of SWIM. (**B**) Top, SWIM in PHA/PHB neuron dendrites. Bottom, kymograph of eGFP::BBS-2 shows single BBSome complexes diffusing across the dendrite (movie S2). Green and red arrows indicate the cilium (c) and soma (s) directions, respectively. (**C**) Top, distance-time plots of all tracked BBSome particles (157 tracks). Bottom, histogram of diffusivity (CVE estimation) shows an average of 0.8 ± 0.1 μm^2^/s. (**D**) Violin plot compares diffusivity of BBSome, IFT-B, IFT-A, IFT-dynein, kinesin-II, and OCR-2–associated vesicles in dendrites. Median and interquartile ranges indicated in violin plots. (**E** and **F**) Kymographs of IFT-B [OSM-6::eGFP (E)] and IFT-A [CHE-11::mCherry (F)] reveal directed transport of vesicle-coated complexes from soma to cilia (movie S3). Green and red arrows indicate cilium (c) and soma (s) directions. (**G**) Histograms show intensities of IFT-B and IFT-A vesicles imaged in dendrites from (E) and (F). (**H**) Histogram of point-to-point velocities of highly directed (α > 1.9) IFT-B (top, 1227 tracks) and IFT-A (bottom, 772 tracks)–coated vesicles with average velocities of 1.74 ± 0.02 and 1.94 ± 0.02 μm/s, respectively. (**I**) Histogram of time lags between subsequent IFT-B (top, 2375 vesicles) and IFT-A (bottom, 1130 vesicles)–coated vesicles. (**J**) Cumulative distribution of time lags, overlayed with least-square fit (black), reveals characteristic times of 5.8 ± 0.5 s for IFT-B (blue) and 8.9 ± 1 s for IFT-A (red). (**K**) Kymograph from dual-color imaging of IFT-B (OSM-6::eGFP; left) and IFT-A (CHE-11::wrmScarlet; middle) in dendrites. Combined image: right, with arrows indicating composition (cyan, only IFT-B; magenta, only IFT-A; and white, both). (**L**) Pie chart, 6.5% of vesicles contain only IFT-A, 26.5% only IFT-B, and 67% both (679 tracks from 8 dendrites). Average value and errors estimated by bootstrapping. Sample statistics are detailed in table S2.

In the PHA/PHB sensory neurons of young adult worms, the cilia are separated from the soma by ~40- to 50-μm-long dendrites (Fig. 1A). To investigate how individual IFT components synthesized in the soma are transported along the dendrite to reach the ciliary base, we visualized the dynamics of individual molecules in the PHA/PHB dendrites using small-window illumination microscopy (SWIM) (*26*). The idea of SWIM is to excite and photobleach only a small region of the sample (5- to 15-μm diameter), allowing continuous and long-term entry of “fresh” proteins, which have not yet been photobleached, into the excited region (Fig. 2A). Using this approach, we observed that BBSome subcomplexes diffuse rapidly in the dendrite (Fig. 2B and movie S2), similar to what we observe for IFT-dynein and kinesin-II (fig. S1A). From these image sequences, we extracted single-particle trajectories, which were used to calculate the diffusion coefficients using a covariance-based estimator (CVE) (*27*). We found that BBSome subcomplexes diffuse faster than IFT-dynein but slower than kinesin-II (Fig. 2, C and D, and fig. S1, B and C; overview of the statistics in table S2). This was expected given that the diffusion coefficient scales with the inverse of the cube root of the mass (*28*), and the BBSome, IFT-dynein, and kinesin-II have masses of ~0.5, ~1.5, and ~0.3 MDa, respectively. Furthermore, this supports our assumption that BBS-2 is incorporated in the BBSome subcomplex and is not diffusing on its own, which would result in a much larger diffusion coefficient given its mass of only ~80 kDa. Our observations suggest that the BBSome and IFT motors do not use active transport for their journey from the soma to cilium along the dendrite but use free diffusion. It can be estimated ($<t>\approx \delta x^{2}/2D$) that transport along the whole length of the ~50-μm-long dendrite would then take on average ~13 min (kinesin-II) to 42 min (IFT-dynein).

We next investigated how IFT-B and IFT-A subcomplexes are transported along the dendrite toward the ciliary base. We observed that both IFT-B and IFT-A, in contrast to the other IFT components, are actively transported in a directed manner from soma to ciliary base (Fig. 2, E and F, and movie S3). Judging from the intensities of the fluorescence spots, multiple IFT-B or IFT-A subcomplexes are transported together, potentially as IFT-B– and IFT-A–coated vesicles. We propose this on the basis of the structural similarity of several IFT-A and IFT-B proteins to COP-I, COP-II, and clathrin proteins (*16*, *17*), which are known to participate in vesicle trafficking outside cilia (*14*). Apart from these vesicles, we also observed that a fraction of the subcomplexes moved diffusively. The diffusion coefficients for this diffusing IFT-B (~2.2 MDa) and IFT-A (~0.8 MDa) subcomplexes are in line with the trend estimated from their molecular weights (Fig. 2D and fig. S1, B and C; statistics in table S2). The IFT-B diffusion coefficient is smaller than that of the other IFT components but substantially higher than that of OCR-2–associated dendritic vesicles [ciliary TRPV-channel protein; fig. S1, B and C; also reported previously (*26*)]. This could indicate that individual IFT-B and IFT-A subcomplexes diffuse, while vesicles containing IFT-B and IFT-A subcomplexes are actively transported. We observed that the fluorescence intensity of these vesicles differed considerably (Fig. 2G), indicating that their size and/or protein content is variable. To obtain quantitative insight in the vesicle transport dynamics, we extracted individual trajectories. Tracked events showed either mostly directed motion, or bouts of directed motion, interspersed with diffusive episodes and pauses (Fig. 2, E and F). To extract the mode of transport, we subjected the trajectories to a windowed mean squared displacement (MSD) approach (*13*, *29*, *30*), which allows determination of the anomalous exponent (α). For directed motion, α is expected to be equal to 2; for diffusive motion, α = 1; and for subdiffusion or pausing α < 1. For IFT-A and IFT-B trajectories that appeared completely directed, we indeed found α values of ~2, while for other trajectories displaying more varied motility, we found α values between 1.5 and 2. After filtering the complete datasets for directed motion (only taking into account time windows when α > 1.9), we determined that the average point-to-point velocity of directed transport is 1.74 ± 0.02 μm/s for IFT-B (average ± error estimated using bootstrapping) and 1.94 ± 0.02 μm/ for IFT-A (Fig. 2H and fig. S2, A to C), which is in the similar range as reported before for OCR-2–associated vesicles (1.99 ± 0.06 μm/s; α > 1.95) (*26*). In the dendrites of *C. elegans* PHA and PHB neurons, where the microtubules are oriented with their minus ends pointing in the direction away for the soma, toward the cilium (*31*), all anterograde dendritic transport is expected to be driven by the minus-end motor cytoplasmic dynein-1. The differences we observed in the velocities of IFT-B– and IFT-A–coated vesicles are consequently most likely not caused by differences in the nature of the motor driving transport but by the number of motors engaged or other properties of the vesicles, like size and membrane properties, or variations in the local architecture of microtubules traveled by the different vesicles. We also considered the time intervals between subsequent vesicles and found that they are exponentially distributed with a characteristic time of 5.8 ± 0.5 s for IFT-B and 8.9 ± 1 s for IFT-A (Fig. 2, I and J). These intervals are substantially shorter than previously reported for OCR-2–associated vesicles (19.8 ± 3.2 s) (*26*), indicating that a diverse pool of vesicles exists that is transported from the soma to the PCMC at different rates. Occasionally, we also observed that IFT-A and, to a lesser extent, IFT-B subcomplexes fall off directed vesicles, which prompted us to measure the time interval between the arrival of subsequent IFT subcomplex–coated vesicles at the PCMC: 7.1 ± 1.1 s for IFT-B vesicles (fig. S2, D and F) and 18.0 ± 2.2 s for IFT-A vesicles (fig. S2, D and F). These numbers suggest that a large fraction of IFT-A subcomplexes dissociates from vesicles before reaching the PCMC.

Last, we imaged IFT-B and IFT-A simultaneously (Fig. 2K and movie S4, OSM-6::eGFP and CHE-11::wrmScarlet) and observed that ~67% of the vesicles contain both IFT-B and IFT-A, ~26.5% only IFT-B, and ~6.5% only IFT-A (Fig. 2L). Since vesicles coated with IFT-A or IFT-B subcomplexes are transported at different rates (Fig. 2J, when studied individually), we performed numerical simulations to explore how this could be possible given that 67% of vesicles carry both IFT-A and IFT-B (see fig. S3 and associated Supplemental Text). We considered whether IFT subcomplex–coated vesicles originate from a single pool (fig. S3, A and B) or from multiple subpools sorted differently at the soma (fig. S3, C and D) and found that the latter model is consistent with the experimental results. From our findings, we hypothesize that IFT subcomplex–coated vesicles, driven from the soma to the PCMC in a directed manner, are derived from multiple overlapping vesicle pools at the soma, each containing varied compositions of IFT-A and IFT-B subcomplexes. In contrast, other protein complexes, including the BBSome and the IFT motors, move diffusively along the dendrite.

### BBSomes bind to PCMC membrane or to assembling IFT trains

**Fig. 3.**
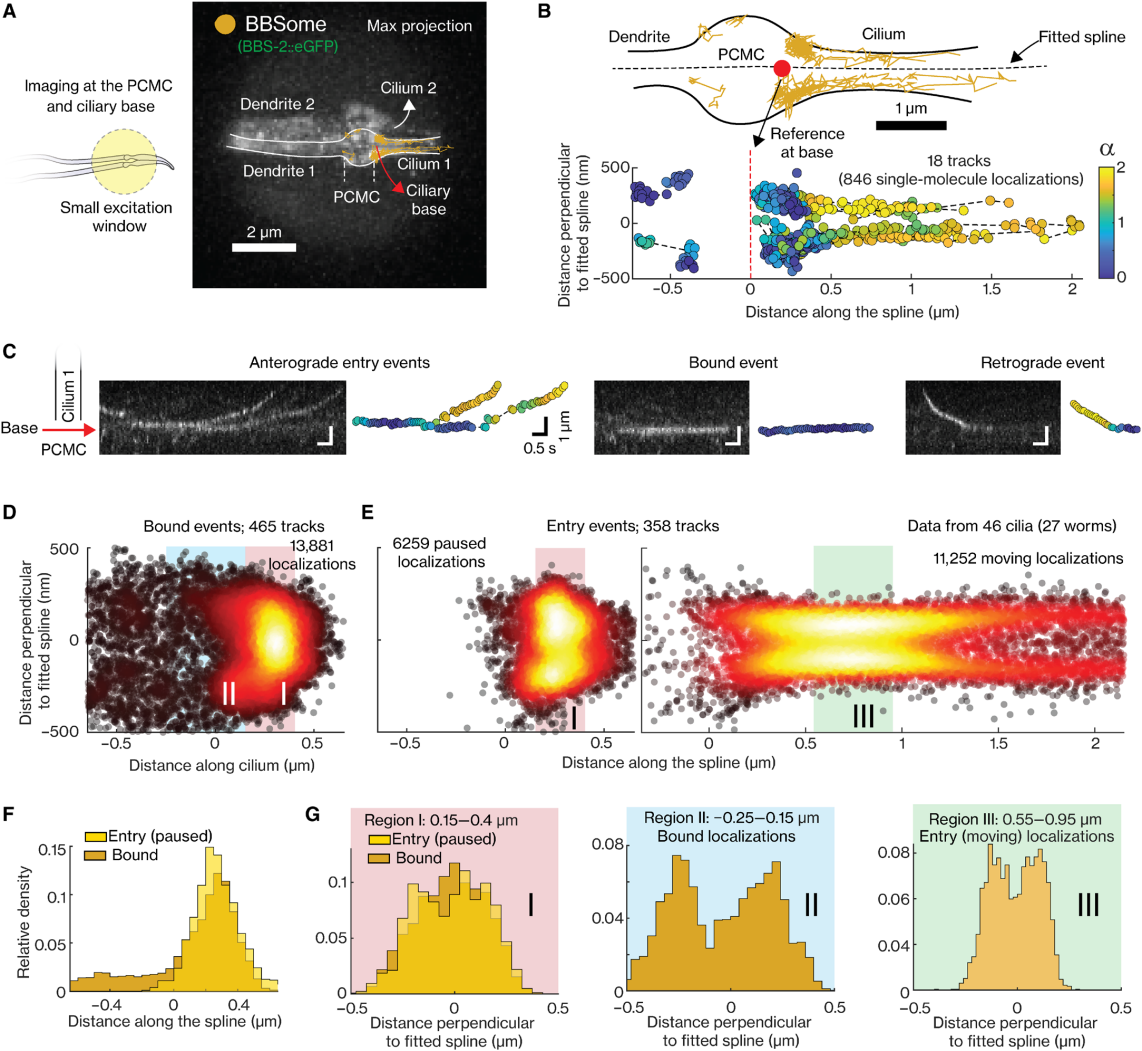
Analysis of the dynamics of individual BBSomes at PCMC and proximal part of the cilium. (**A**) Right, maximum projection of an example movie (movie S5) shows the section of dendrites, PCMC, and cilia of a PHA/PHB neuron pair illuminated by a small excitation window (left, illustration), where the dynamics of eGFP::BBS-2 (BBSome) is visualized. (**B**) Top, tracked single-molecule events with the outline of the cell indicated. Dotted black line displays the fitted spline, and the red point indicates the reference point at the base. Bottom, the tracked events plotted in ciliary coordinate. The color of the localizations indicates the α value, a measure of degree of directedness of the event at that localization. α value is ~2 for purely directed transport and ~1 for purely diffusive transport. (**C**) Kymographs displaying example anterograde entry events (left), event bound at the ciliary base (middle), and retrograde event (right) with corresponding tracks (color of localizations indicate the α). (**D** to **E**) Superresolution map of single-molecule localizations obtained 465 tracks of PCMC-binding events [(D) *N* = 13,881 localizations] and 358 tracks of entry events [(E) left, *N* = 6259 paused localizations (α < 1); right, *N* = 11,252 moving localizations (α > 1.2)] obtained from 46 cilia. (**F**) Distribution of distance along the cilium for localizations of PCMC-binding events and paused localizations of entry events. (**G**) Distribution of distance perpendicular to ciliary spline for localizations of PCMC-binding events and paused localizations of entry events (α < 1) between 0.15 and 0.4 μm (left, region I), bound events between −0.25 and 0.15 μm (center, region II), and moving localizations of entry events between 0.55 and 0.95 μm (right, region III). Regions I, II, and III are indicated in [(D) and (E)]. Overview of sample statistics in this figure is provided in table S3.

We next used SWIM to investigate the dynamics of individual IFT-train subcomplexes in the region where the ciliary base meets the dendrite (Fig. 3A, left), focusing first on BBSomes (Fig. 3A, right, and movie S5). The BBSome tracks showed three distinct kinds of behaviors. (i) In some tracks, BBSomes switched from a diffusive to an immobile state close to the ciliary base, followed by a short pause, before they started to move into the cilium, speeding up beyond the TZ (Fig. 3C, left). These tracks are similar to those we observed before for IFT motors entering the cilium (*13*), and we infer that they represent BBSome subcomplexes that bind to immobile, assembling IFT trains at the ciliary base (*12*) and are transported into the cilium after train assembly has completed. We refer to these tracks as “ciliary-entry events.” (ii) In other tracks, BBSomes switched from a diffusive to an immobile state apparently binding to the PCMC membrane, in most cases close to the ciliary base (Fig. 3C, middle). In these tracks, we did not observe ciliary entry, but we often saw BBSomes being released again from their static location and diffusing in the dendrite. In other cases, BBSomes appeared to start diffusing again while remaining attached to the PCMC membrane. These latter particles appeared to diffuse substantially faster and often “hopped” to another static location in the PCMC. We refer to this second class of track as “PCMC-binding events.” It has been shown before that BBSomes can be recruited to the membrane near the ciliary base by ARF-like GTPase ARL6/BBS3 (*32*–*34*). We thus propose that the PCMC-binding events represent BBSomes freely diffusing in the dendrite that stochastically bind to the PCMC membrane. (iii) In the few retrograde tracks we observed, BBSomes appeared to exit the cilium and subsequently remained stuck in the PCMC (Fig. 3C, right). As SWIM primarily captures anterograde events in the illuminated region, retrograde events are observed only very rarely and are therefore excluded from the subsequent analysis.

To obtain a more detailed spatial picture of paused BBSome localizations at the ciliary base, we extracted superresolution fluorescence maps from the tracks of the PCMC-binding and ciliary entry events (Fig. 3, D and E left, respectively; statistics in table S3). To pool information from multiple cilia in different worms, we transformed the track coordinates (*x*_*i*_, *y*_*i*_) to ciliary coordinates ($c_{∥\_i}$, $c_{⊥\_i}$; see Methods; Fig. 3B and fig. S3A). Further, we filtered the tracks using the windowed MSD approach for α ≤ 1, (see example tracks in Fig. 3B). The distribution of paused localizations from the PCMC-binding tracks shows a maximum at the ciliary base (at ~0.3 μm), but with a long tail into the PCMC (Fig. 3F). The distribution of paused localizations from the ciliary-entry events also shows a sharp peak, slightly shifted toward the dendrite (at ~0.2 μm; Fig. 3F) but without the long tail into the PCMC. The distribution of the moving localizations of BBSome entry events (α > 1.2; Fig. 3E, right) looks similar to those we observed before for the IFT motors (*13*, *35*), revealing the characteristic shape of the initial part of the cilium: relatively broad at the ciliary base, tapering at the TZ, and “bulging” out at the proximal segment of the cilium. We next looked into the distributions perpendicular to the ciliary long axis. For localizations of ciliary entry as well as PCMC-binding events in region II (Fig. 3G), axial distributions show two maxima equidistant from the center. Before (*13*), we have interpreted the bimodal distributions to be due to the two-dimensional (2D) projection (by our imaging approach) of the 3D distribution along a hollow cylinder (the axoneme or the PCMC membrane); see also fig. S3B. For the PCMC-binding events in region I (close to the ciliary base), it is likely that the narrow membrane width in this region does not allow us to discern the two maxima and only a single maximum at the center is observed (Fig. 3G, left). Together, single-particle tracking reveals that BBSome subcomplexes show two distinct behaviors at the ciliary base after exiting the dendrite: One population of BBSomes docks to the PCMC membrane, primarily close to the ciliary base, while the other attaches to assembling IFT trains at the ciliary base. The overall single-particle localization maps are consistent with the ensemble intensity profiles at the PCMC and proximal part of the cilia (Fig. 1F).

### IFT-A– and IFT-B–coated vesicles are sorted differently near the ciliary base

**Fig. 4.**
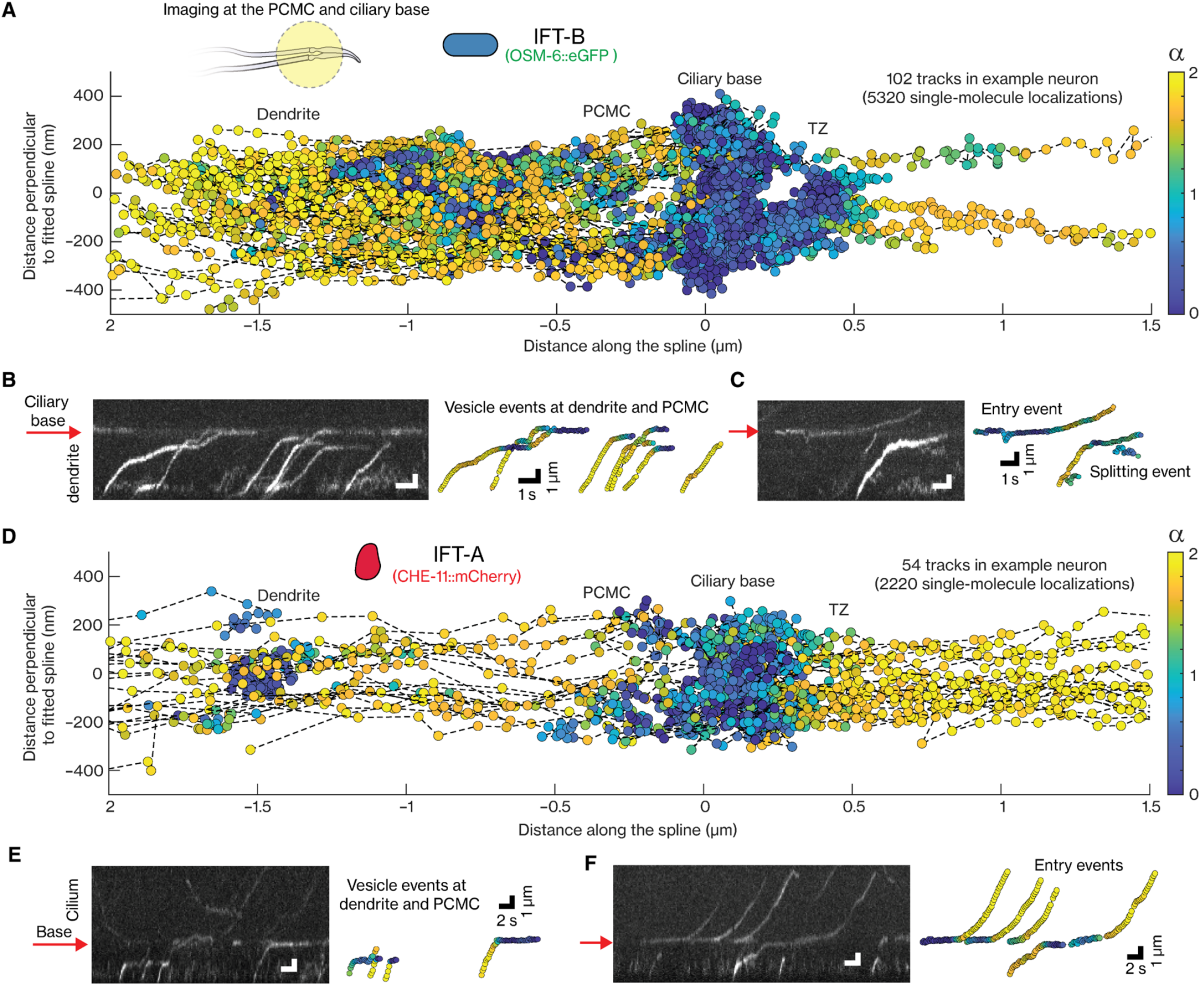
Dynamics of individual IFT-B and IFT-A complexes at PCMC and ciliary base. (**A**) Single-particle IFT-B tracks obtained from example OSM-6::eGFP–labeled worm imaged using SWIM (movie S6), plotted in cilia coordinates. Inset, illustration of section of dendrites, PCMC, and cilia of PHA/PHB neuron pair illuminated using SWIM (left, illustration). (**B** to **C**) Representative kymograph showing example IFT-B events (left) and corresponding tracks (right). (B) Directed IFT-B–coated vesicles moving from the dendrite into the PCMC. These vesicles slow down or pause at the dendrite end of PCMC (~1 μm from ciliary base), with individual IFT-B complexes eventually bleaching, diffusing, or reaching ciliary base in a directed manner where they pause for a longer time. (C) Event on left, an IFT-B complex, diffusing in dendrite, docks at ciliary base, hops to another location at base, and enters the cilium after a pause. Event on right, a high-intensity IFT-B–coated vesicle moving from dendrite toward the cilia, releasing IFT-B complexes that diffuse. (**D**) Single-particle IFT-A tracks obtained from an example CHE-11::mCherry–labeled worm (movie S7) plotted in the cilia coordinates. (**E** to **F**) Representative kymograph showing example IFT-A events (left) and corresponding tracks (right). (E) Directed IFT-A–coated vesicles moving from dendrite into PCMC. While most IFT-A complexes bleach or switch to a diffusive state as IFT-A–coated vesicles move through PCMC, some reach the ciliary base where they pause for a long duration (rightmost event). (F) IFT-A complexes, diffusive in PCMC, dock at ciliary base and enter the cilium. Occasionally IFT-A complexes reaching the base coated on directed vesicles directly enter the cilia (rightmost event). Red arrows in all kymographs indicate the location of the ciliary base. Scale bar of kymograph (left) are same as scale bar for corresponding tracks (right). Color of localizations in all plots indicates the α value.

Next, we imaged the dynamics of IFT-B arriving from the dendrite into the PCMC (see example neuron-pair in movie S6A and tracked single-molecule localizations from one neuron in Fig. 4A). We observed that IFT-B–coated vesicles moved directedly and apparently actively from the dendrite toward the PCMC, where they slowed down or paused briefly between −1.1 and −0.7 μm from the ciliary base (example events in movie S6B and Fig. 4B). Part of the vesicles appeared to continue their directed route, docking at the ciliary base, where they paused, often for several seconds, occasionally hopping from one location to another. Some vesicles did not seem to reach the ciliary base because the fluorescence signal disappeared at the pause location close to the dendrite-PCMC junction, either by photobleaching or by IFT-B subcomplexes dissociating from the paused IFT-B–coated vesicles and diffusing away (as could be observed occasionally: Fig. 4C and movie S6B). Collectively, we refer to these events as “vesicle” events. We also observed diffusive IFT-B docking at the ciliary base, pausing for a while, before entering the cilium (Fig. 4C). This behavior is characteristic of “entry events”: IFT components associating with an assembling IFT train that enters the cilium after the train starts moving [like we have observed before for BBSomes (Fig. 3C) and other IFT components (*13*)]. We did not observe direct entry of IFT-B subcomplexes from coated vesicles that had been transported toward the ciliary base and paused there. It might be that such entry events are rare since most complexes photobleach during the relatively long pause at the base or since other vesicles or diffusive complexes arrive in the vicinity, making it difficult to distinguish them reliably as single events. Apart from vesicle and entry events, we also obtained “stuck” events, which are due to diffusive particles docking at the ciliary base, where they remain static until the signal disappears because of photobleaching or gradual dissociation of IFT-B.

Also, IFT-A displayed rich dynamics near the PCMC and the ciliary base (Fig. 4D and movie S7A), with some key differences to IFT-B. Since IFT-A dissociates from vesicles more readily than IFT-B (fig. S2, D and F), a large fraction of IFT-A does not reach the ciliary base. The pool of IFT-A–coated vesicles, where IFT-A subcomplexes remain attached, did not seem to pause near the dendrite-PCMC junction but slightly before, toward the dendrite, mostly around −1.5 μm from the base. IFT-A entry events, where IFT-A subcomplexes from a cytosolic pool dock at the ciliary base and, after a short pause, move into the cilia, were more frequently observed than in the case of IFT-B (Fig. 4, A, D, and E). Partly, this is due to vesicles containing much fewer IFT-A by the time they reach the ciliary base, making the ciliary base far less busy and single particles far more straightforward to discern. Furthermore, on rare occasions, we also observed ciliary-entry events arising directly from an IFT-A–coated vesicle parked at the ciliary base (Fig. 4F), suggesting that it is possible for IFT-A to swiftly associate with assembling IFT trains from coated vesicles.

**Fig. 5.**
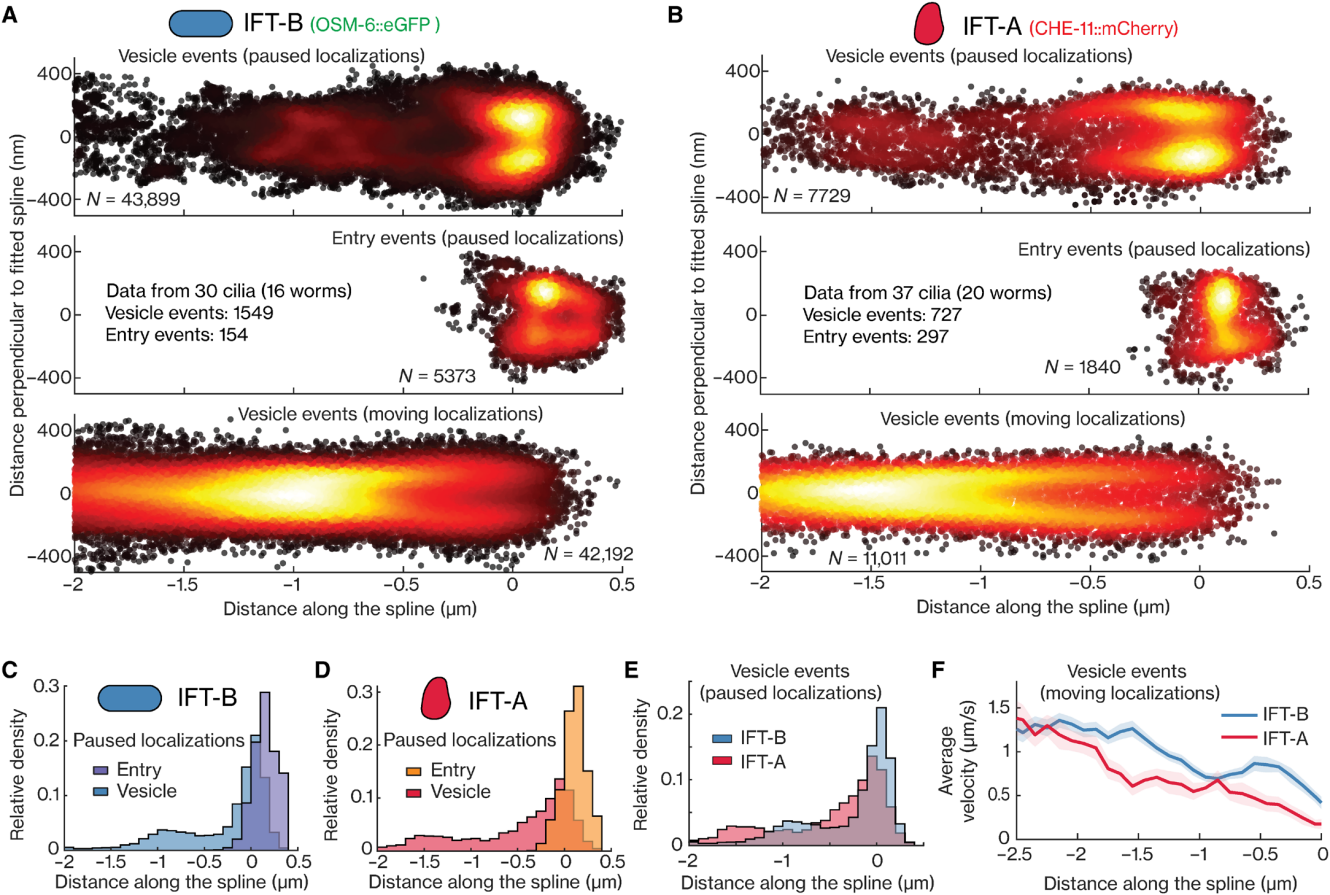
Single-particle localizations reveal that IFT-B and IFT-A are sorted differently in the PCMC and ciliary base. (**A** and **B**) Superresolution map of single-particle localizations obtained from tracks of IFT-B and IFT-A. (A) Localizations of IFT-B from 30 cilia. Top, paused localizations from vesicle events (43,899 localizations from 1549 tracks); middle, paused localizations from entry events (5373 localizations from 154 tracks); bottom, moving localizations from vesicle events (42,192 localizations). (B) Localizations of IFT-A from 37 cilia. Top, paused localizations from vesicle events (7729 localizations from 727 tracks); middle, paused localizations from entry events (1840 localizations from 297 tracks); bottom, moving localizations from vesicle events (11,011 localizations). (**C** to **E**) Distributions of distance along fitted spline for paused localizations: (C) from vesicle and entry events of IFT-B; (D) from vesicle and entry events of IFT-A; (E) from vesicle events of IFT-B and IFT-A. (**F**) Velocity distribution between −2.5 and 0 μm along the fitted spline, obtained from moving localizations of vesicle events, for IFT-B (blue) and IFT-A (red). Overview of sample statistics in this figure is provided in table S2.

To obtain a more detailed spatial picture of IFT-A and IFT-B arriving at the ciliary base, we generated superresolution fluorescence maps from the localizations of IFT-B (Fig. 5A) and IFT-A (Fig. 5B) in vesicle and ciliary entry tracks obtained from multiple cilia and worms using our windowed MSD approach to filter for paused (α < 1) and moving (α > 1.2) localizations (statistics in table S3). The distribution of paused localizations of entry events peaks closer to the TZ (peak at ~0.2 μm) than that of paused localizations of vesicle events (peak at ~0 μm), both for IFT-B (Fig. 5C) and IFT-A (Fig. 5D). This indicates that vesicles stop moving at slightly different locations close to the ciliary base from where single IFT-B and IFT-A subcomplexes associate with assembling IFT trains. The distribution of localizations of stuck events is in between those of paused entry and vesicles localizations (distributions of IFT-B events in fig. S4A), suggesting that they are a combination of entry events that photobleach before the train has moved into the cilium and diffusive IFT-B–coated vesicles binding at the ciliary base. We observed a second peak in the distribution of paused vesicle localizations at the dendrite-PCMC junction for IFT-B at ~−0.9 μm and slightly further in the dendrite for IFT-A at ~−1.5 μm (Fig. 5E), as is also evident in the example trajectories shown in Fig. 4. Also, the distributions of moving IFT-B and IFT-A vesicle localizations show secondary peaks at ~−0.9 and ~−1.5 μm, respectively (Fig. 5, A and B, bottom). Together, these superresolution maps indicate that IFT-A– and IFT-B–coated vesicles heading toward the ciliary base initially pause briefly at distinct locations, with IFT-B vesicles primarily pausing near the dendrite-PCMC junction (at ~−0.9 μm) and IFT-A vesicles pausing throughout the dendritic region near the PCMC and at the PCMC (peaking at ~−1.5 μm). Further support for this can be found in the local, averaged point-to-point, axial velocities (Fig. 5F), which show that IFT-A–coated vesicles slow down earlier (from ~−2 μm) than IFT-B–coated vesicles (from ~−1.5 μm). Furthermore, the axial distributions of moving IFT-A and IFT-B in the PCMC appear hollow with substantially more localizations along the periphery than in the center (fig. 5, A and B, bottom), indicating that the microtubules extending from the dendrite into the PCMC, along which the IFT-coated vesicles are transported, are organized along the PCMC membrane. Overall, our observations reveal that IFT-B– and IFT-A–coated vesicles are differently sorted, pausing at different locations before and at the ciliary base. At these pause locations, IFT-B and IFT-A subcomplexes gradually dissociate from the vesicles, forming a diffusive pool in the PCMC. It is mostly IFT-B and IFT-A subcomplexes from this diffusive pool that associate with assembling IFT trains at the ciliary base. Once the assembly of these trains is completed, they ferry the IFT-B and IFT-A subcomplexes into the cilium.

### IFT-train subcomplexes show different dynamics during ciliary entry

**Fig. 6.**
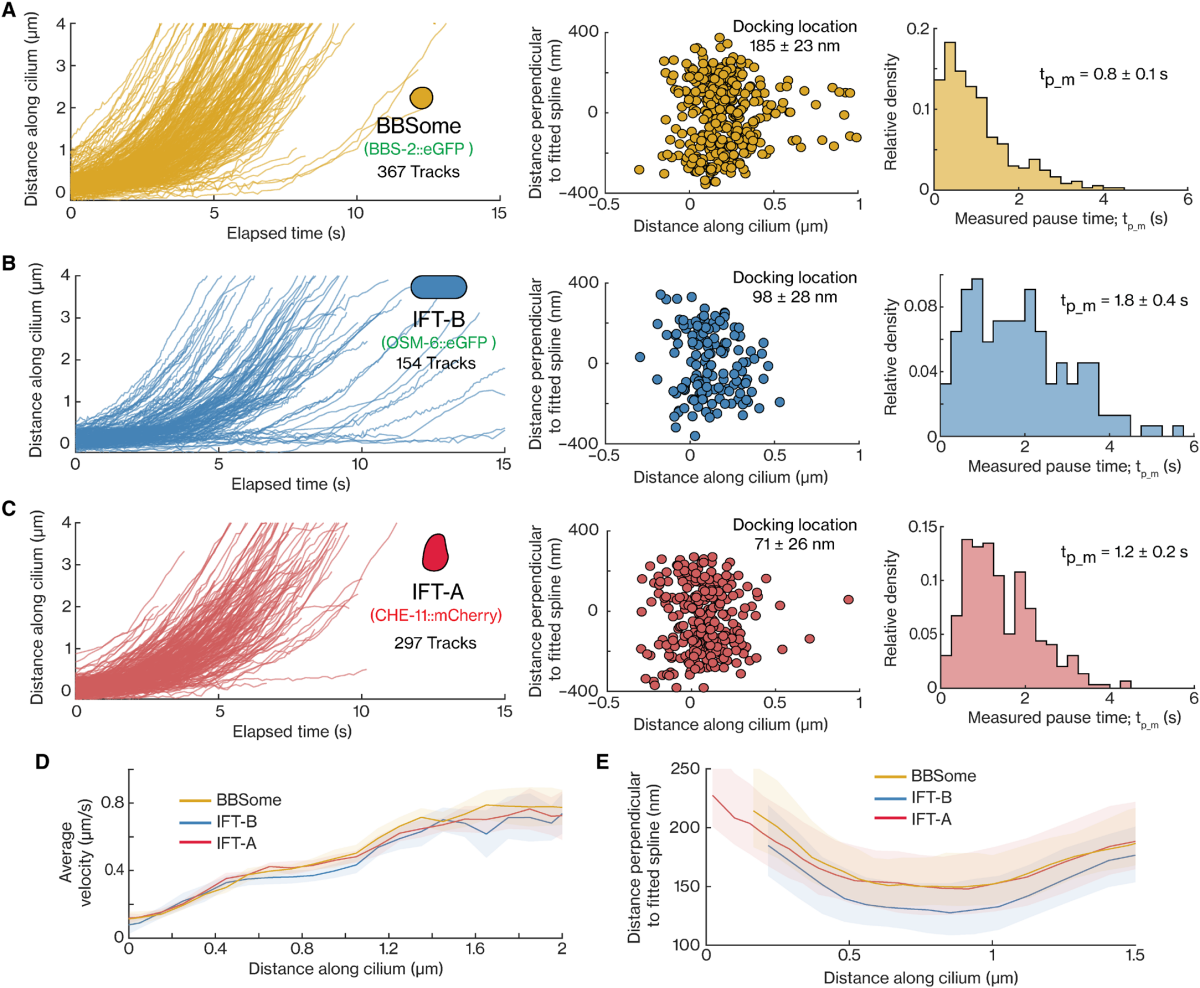
Dynamics of individual BBSome, IFT-B, and IFT-B complexes entering cilia. (**A** to **C**) Distance-time plots (left), distribution of docking locations (middle), and distribution of measured pause time (right) for IFT train complexes: (A) 367 entry tracks of BBSome (BBS-2), average docking location 185 ± 23 nm, average measured pause time is 0.8 ± 0.1 s; (B) 154 entry tracks of IFT-B (OSM-6), average docking location 98 ± 28 nm, average measured pause time is 1.8 ± 0.4 s; (C) 297 entry tracks of IFT-A (CHE-11), average docking location 71 ± 26 nm, average measured pause time is 1.2 ± 0.2 s. (**D**) Distribution of binned average velocities (solid line) along the length of the cilium for BBSome (yellow), IFT-B (blue), and IFT-A (red). (**E**) The shape of the distribution, binned along the ciliary length, obtained from moving single-molecule localizations of entry events corresponding to BBSome (yellow), IFT-B (blue), and IFT-A (red). Shaded areas in [(D) and (E)] indicate the errors. Average values and errors are estimated using bootstrapping. Overview of sample statistics in this figure is provided in table S3.

Last, we compared BBSome, IFT-B, and IFT-A entry events (Fig. 6, A to C, left). In all these tracks, we observed that the IFT components, mostly part of a diffusive pool in the PCMC, dock at the ciliary base, presumably by attaching to assembling IFT trains, to pause for a brief interval of time, before moving into the cilium (Fig. 6, A to C, left). We found that the distributions of docking locations are different for the different IFT-train subcomplexes: While most BBSomes dock onto IFT trains between 0 and 0.4 μm from the ciliary base (average 0.19 ± 0.03 μm), some events start deeper inside the TZ (0.5 to 1 μm). In contrast, IFT-A and IFT-B dock almost exclusively between 0 and 0.3 μm [averages 0.10 ± 0.03 and 0.07 ± 0.03 μm, respectively (Fig. 6, A to C, middle)]. Similar to this observation, the distributions of paused localizations of BBSome, IFT-B, and IFT-A entry events also highlight that, on average, BBSomes are statically associated with IFT trains slightly closer to the TZ than IFT-B and IFT-A (fig. S4). For each entry track, we also determined the measured pause time, $t_{p\_m}$, defined as the duration it takes a single complex to move 100 nm along the cilium. We found that $t_{p\_m}$ is substantially smaller for BBSomes (0.8 ± 0.1 s, average ± error estimated using bootstrapping; Fig. 6A, right) than for IFT-A (1.2 ± 0.2 s; Fig. 6C, right), and IFT-B (1.8 ± 0.4 s; Fig. 6B, right). We note that these measured pause durations are highly affected by eGFP/mCherry photobleaching, which terminates many trajectories before they can be classified as entry events. To account for this, we use simulations [see fig. S5A and Methods for details; as performed before for estimating pause times of IFT motors (*13*)]. These simulations allow us to estimate the actual pause times of BBSomes to be ~0.4 to 0.7 s, of IFT-B > 9 s (fig. S5F; example simulation in fig. S5E), and of IFT-A ~2 to 11 s (fig. S5H; example simulation in fig. S5G; we note here that the estimation of the actual pause duration becomes relatively inaccurate when the measured pause time is similar to the characteristic photobleaching time). These estimates reveal that BBSomes pause for, on average, a much shorter duration than IFT-A and IFT-B subcomplexes before ciliary entry. From the moving localizations in these entry tracks, we obtained the average point-to-point velocity profiles, which were very similar for the three IFT train complexes (Fig. 6D) as well as IFT-dynein (*13*), with the velocity increasing to ~0.4 μm/s in the first ~0.6 μm of the TZ and then remaining constant until ~1.1 μm before increasing steeply after ~1.1 μm, where the TZ ends. Last, we estimated the diameter of the 3D cylinder, along which the moving IFT components are distributed, and found that BBSomes and IFT-A subcomplexes localize further away from the longitudinal axis of the ciliary axoneme than IFT-B (Fig. 6E). This observation correlates with studies that reveal that IFT-A and BBSome interact with ciliary membrane proteins (*1*, *36*) and would structurally require to face the ciliary membrane. IFT-B, on the other hand, was located closer to the axoneme, to which it is connected via kinesin-2 motors. Thus, our observations indicate that IFT-B and IFT-A associate with assembling IFT trains during the early stages of assembly when they are immobile, while BBSomes associate with IFT trains at the final stages of assembly or already assembled trains moving inside the cilium. Furthermore, IFT-train components have the same characteristic velocity profile since they move together across the TZ to enter the cilium. Single-molecule localization maps hint toward close association of the BBSome and IFT-A with the ciliary membrane.

## DISCUSSION

In most ciliated systems, the cilia protrude directly from the cell soma, which makes it challenging to visualize the dynamics of ciliary proteins in the cell soma and near the ciliary base. In contrast, in the chemosensory neurons of *C. elegans*, the soma is separated from the cilium by a long dendrite, tens of micrometers long, similar to mammalian (*37*) and *Drosophila* olfactory neurons (*38*). We used this particular geometry in PHA/PHB neurons, using SWIM (*26*), to selectively illuminate (and photobleach) a small section of the neuron and observe how different IFT components move across the illuminated section. Using this approach, we visualized how IFT components traverse from the soma to the ciliary base and how they assemble into anterograde IFT trains at the ciliary base to enter the cilium.

### Diffusion plays crucial role in transporting intact IFT-train subcomplexes across dendrite

**Fig. 7.**
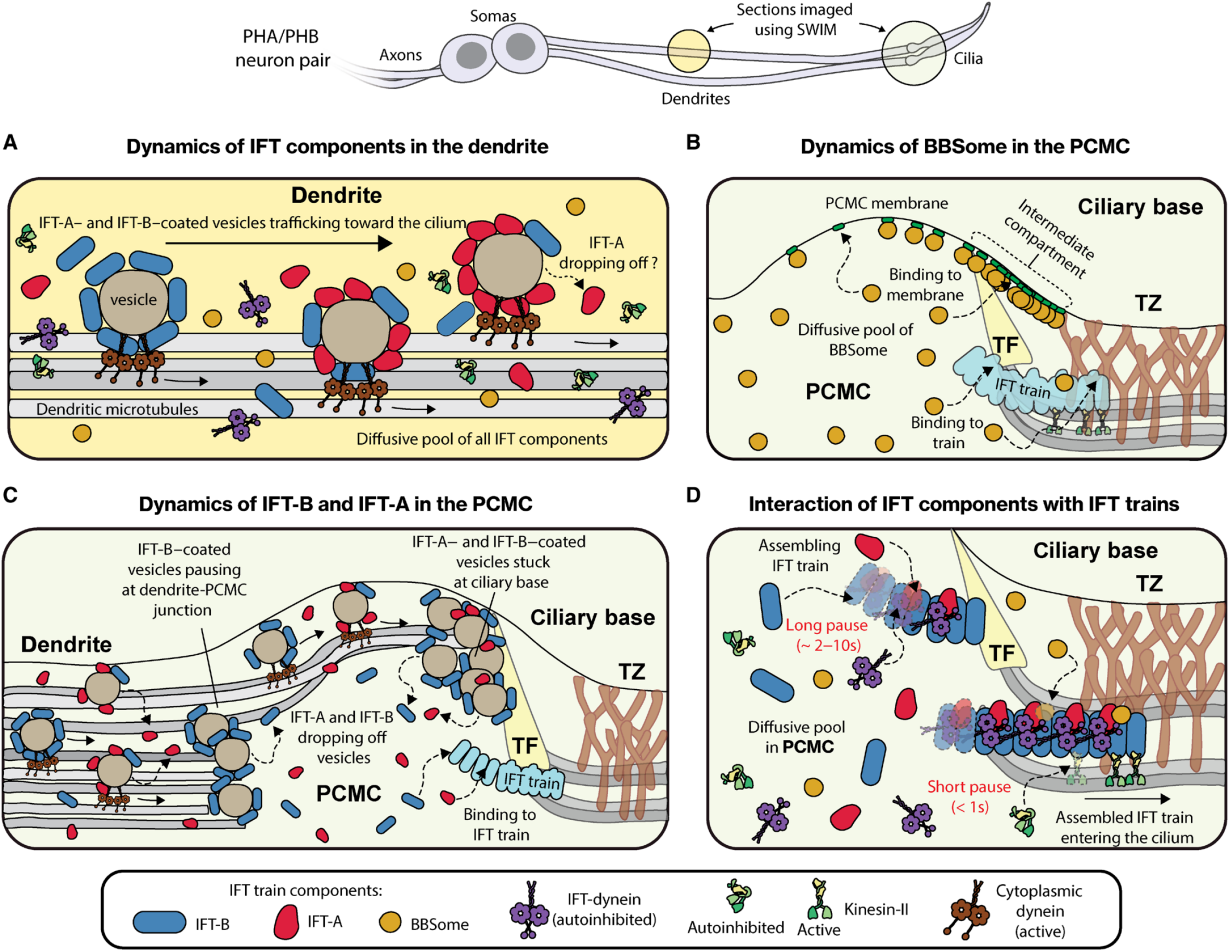
Illustration of the dynamics of IFT components as they traffic across the dendrite, organize at the PCMC, and enter the cilium. (**A**) BBSomes, IFT-dynein, and kinesin-II move across the dendrite purely via diffusion, while IFT-B and IFT-A are transported in a directed manner via vesicles. (**B**) The diffusive pool of BBSomes at the PCMC either associates with anterograde IFT trains parked at the ciliary base or with the PCMC membrane, primarily in the periciliary barrier (region between the TZ and the TF). (**C**) The dynamics of IFT-A– and IFT-B–coated vesicles at the PCMC. IFT-B–coated vesicles stall temporarily at the dendrite-PCMC junction before parking for a much longer duration at the ciliary base. IFT-A–coated vesicles also park at the ciliary base but do not stall at the dendrite-PCMC junction. Individual IFT subcomplexes fall off the vesicles as they move toward the ciliary base. IFT-A dissociates from vesicles more frequently than IFT-B, resulting in a much larger pool of vesicle-associated IFT-B than IFT-A at the ciliary base. (**D**) IFT components load onto assembling anterograde IFT trains from a diffusive pool in the PCMC. BBSome and kinesin-II associate with IFT trains only during the late stages of assembly, while IFT-A, IFT-B, and IFT-dynein associate with IFT trains throughout the period of assembly.

First, we investigated whether IFT proteins reach the ciliary base by random diffusion or whether they are specifically transported there by active transport. We found that all investigated IFT components undergo diffusion in the dendrite, with BBS-2 (BBSome) and IFT motors moving exclusively via diffusion, whereas OSM-6 (IFT-B) and CHE-11 (IFT-A) also being transported in a directed manner, as part of the vesicles (discussed later). The diffusion coefficients we extracted for BBSome, IFT-A, and IFT-B subunits diffusing in the dendrites are consistent with these subunits being part of the respective (BBSome, IFT-A, and IFT-B) subcomplexes and being transported as intact subcomplexes (Fig. 7A). This observation is in line with several earlier findings. It has been shown before that (i) functionally related IFT proteins show similar FRAP recovery rates at the ciliary base, indicating shared recruitment dynamics (*10*, *11*); (ii) some IFT proteins cannot be expressed or purified without their subcomplex partners (*39*, *40*); and (iii) IFT proteins mislocalize when subcomplex partners are mutated (*10*, *41*). The IFT-B complex is composed of two subcomplexes, IFTB1 and IFTB2, which showed slightly different recruitment dynamics in a FRAP study (*10*). While in this study, we assume that OSM-6 represents dynamics of complete IFT-B subcomplexes, it needs to be tested whether IFT-B1 and IFT-B2 always form a complex in the dendrite and cilium.

### BBSomes at ciliary base associate with PCMC membrane or IFT trains

Visualization of the single-particle dynamics of BBSomes diffusing near the ciliary base revealed that they either associate with the PCMC membrane or bind to assembling anterograde IFT trains that, after assembly is completed, transport the BBSomes into the cilium (Fig. 3, A to D, and Fig. 7B). Membrane-associations occur throughout the PCMC but are enriched proximal to the TZ in an ~300-nm-wide region (Fig. 3D). It has been shown before that BBSomes are recruited to the membrane by ARL-6/BBS-3, switching from a closed autoinhibited state in solution to an open membrane-bound conformation (*32*, *33*). ARL-6/BBS-3 was found to be enriched at the ciliary base, close to the transitional fibers in mammalian cells (*42*, *43*) and *Chlamydomonas* (*44*). Furthermore, recent studies have revealed that BBSomes and ARL-6/BBS-3 associate with retrograde IFT trains to remove activated G protein–coupled receptors (GPCRs) from the cilia (*45*). The activated GPCRs are driven across the TZ into an intermediate compartment located between the TZ and a nearly impassable periciliary barrier, where they reside before being recycled into the cilium (*36*, *45*). On the basis of our observations and these previous findings, we hypothesize that, in *C. elegans*, BBSomes are recruited to the PCMC membrane by ARL-6/BBS-3, which is likely enriched at the proposed intermediate compartment. We also observed that retrograde-moving BBSomes cross the TZ and localize in the membrane-bound pool (Fig. 3C), suggesting that BBSomes plays a key role in ciliary gating of membrane proteins at the ciliary base. To our surprise, BBSomes entering the cilium pause only for a short duration and the localization profile of the entering BBSome is distinct from the membrane-bound pool (Fig. 3, D to F). This suggests that BBSomes associating with IFT trains directly originate from the cytosolic pool and not from the ARL-6/BBS-3–associated membrane-bound pool. In *Chlamydomonas* it has been shown that ARL-6/BBS-3 is required for recruiting BBSomes to the basal body but not for ciliary entry (*46*). This suggest that BBSomes are present in two separate pools at the ciliary base: (i) a membrane-bound pool primarily localized between the TZ and the transition fibers, which likely forms the periciliary barrier (*36*, *45*) and (ii) a pool associating with stationary anterograde trains assembling at the ciliary base. Further single-particle studies of the BBSome in conjunction with ARL-6/BBS-3 and transition fiber proteins, like FBF-1, could reveal a more detailed picture on the functional role of BBSomes at the ciliary base.

### IFT subcomplexes carried on vesicles to PCMC, dissociate, forming a diffusive pool

In contrast to BBSomes and IFT motors, IFT-A and IFT-B are transported in an active manner from the soma to the PCMC, most likely, in the form of vesicles coated with these IFT subcomplexes (Fig. 2, D to I, and Fig. 7A), with previous studies also showing that several IFT-B and IFT-A proteins cluster around periciliary vesicles (*18*–*21*). Furthermore, in *C. elegans*, several ciliary transmembrane proteins like OCR-2 (*26*, *29*) and OSTA-1 (*47*), as well as cytosolic proteins like guanylyl cyclase GCY-12 (*48*) and tubulin glutamylase TTLL-11 (*49*) have been shown to undergo directed vesicular trafficking across the dendrite, and these periciliary vesicles appear to accumulate in the PCMC (*50*, *51*). Thus, diverse cellular components are targeted in a specific manner to dendritic ends, transported at different rates [whenever characterized (*26*, *48*)], with IFT proteins likely acting as vesicular COPs. On this basis, we hypothesize that IFT-B and IFT-A proteins play a key extraciliary role in selective sorting and transport of proteins to the PCMC, a functional role that might be universal to all ciliated systems and which can be conveniently visualized in our system because of the distinct geometry of the ciliated sensory neurons in *C. elegans.*

We observed that the dynamics of IFT-A– and IFT-B–coated vesicles entering the PCMC are not completely the same. IFT-B–coated vesicles often paused briefly at the dendrite-PCMC junction, located ~0.9 μm from the ciliary base, before moving to the ciliary base where they stalled for a longer duration (Fig. 7C). The PCMC membrane is known to be a separate compartment from the dendrite (*52*) and is enriched with cilium-related sensory signaling proteins as well as regulators of endocytosis and membrane trafficking (*50*, *53*, *54*). The mechanism of this compartmentalization is not well understood, and it is unclear whether IFT-B–coated vesicles perform a functional role while stalling at the compartment junction. The final “parking” destination for most IFT-B–coated vesicles is adjacent to the TZ at the ciliary base (*55*). We note that the parking location of the vesicles is on the dendritic side of where the membrane-bound pool of BBSome localizes (fig. S5, A and B), suggesting that these vesicles cannot cross the periciliary barrier formed by the transition fibers (*5*, *45*). Furthermore, this parking location of IFT-B–coated vesicles is slightly proximal to the region where individual IFT-B subcomplexes associate with stationary IFT trains (Fig. 5, A to D). In line with this observation, in *Chlamydomonas*, IFT-B has been shown to have distinct anterior and posterior pools at the ciliary base, with IFT-A primarily colocalizing with only the anterior pool (*56*). This suggests that the organization of IFT-B subcomplexes at the ciliary base into two distinct pools, corresponding to periciliary vesicles and assembling IFT trains, might be similar in *C. elegans* and *Chlamydomonas*. In contrast to IFT-B, a major fraction of IFT-A dissociates from the vesicles before arriving near the ciliary base, with the vesicles still associated with IFT-A stalling at the dendrite (around the region ~1.5 μm from the ciliary base), along the PCMC membrane and at the ciliary base. In dual-color experiments, IFT-A and IFT-B colocalized in most vesicles, although vesicles containing exclusively IFT-A or IFT-B were also observed. Our numerical simulations, to explore whether IFT subcomplex–coated vesicles originate from multiple pools (fig. S3), suggest that there is likely a rich compositional diversity of periciliary vesicles, and it is plausible the IFT-A and IFT-B proteins selectively sort vesicles at different locations in the PCMC. We also observed that both IFT-B– and IFT-A–coated vesicles move along the periphery of the PCMC, indicating that the dendritic microtubules extending into the PCMC are organized along the PCMC membrane and likely facilitate the interaction of periciliary vesicles with the membrane.

We observed that individual IFT-A and IFT-B subcomplexes detach from IFT-coated vesicles moving into the PCMC from the dendrite, forming a pool of IFT subcomplexes diffusing in the PCMC (Fig. 7C), as well as the dendrite (Fig. 7A). IFT-B subcomplexes mostly dissociate from vesicles after they stall at the ciliary base while IFT-A subcomplexes rapidly detach from vesicles while they are moving toward the ciliary base. This results in the substantial enrichment of vesicle-bound IFT-B at the ciliary base in comparison to IFT-A, as observed in the ensemble fluorescence-intensity profiles (relative to the pool in the TZ; Fig. 1G). IFT-B and IFT-A entry events primarily originate from the diffusive pool of IFT subcomplexes. We never observed IFT-B entry events directly originating from vesicles stalled at the ciliary base, although on rare occasions, IFT-A originating from vesicles does appear to enter the cilium (Fig. 4F). A recent structural study has shown that the IFT-A domains required to coat periciliary vesicles are shielded when IFT-A polymerizes on IFT trains (*57*), suggesting that IFT-A cannot bind with vesicles and IFT trains at the same time. Insights on IFT-B in this regard are still lacking.

Overall, our findings suggest that IFT-train subcomplexes enter the cilium mostly from a diffusive PCMC pool, replenished with individual IFT-train subcomplexes that dissociate from continuously arriving IFT-B– and IFT-A–coated vesicles. This supply helps to maintain a concentration gradient of IFT subcomplexes, decreasing from the PCMC to the soma, with the frequency of vesicle arrivals regulating the concentrations and the rate of fresh IFT complexes associating with assembling trains.

### IFT components associate with assembling IFT trains in subsequent stages

We observed that individual BBSome, IFT-B, and IFT-A subcomplexes enter the cilium after first pausing for a while, apparently being incorporated into immobile, assembling IFT trains at the ciliary base (Fig. 7D), similar to what we observed before for IFT-dynein (*13*). In *Chlamydomonas*, FRAP and electron microscopy studies have shown that IFT trains take ~9 s to assemble at the ciliary base, where anterograde trains at various assembly stages align at the TZ, with IFT-A and IFT-dynein oligomerizing linearly on an IFT-B scaffold (*11*, *12*). This is in line with our finding that, on average, IFT-B pauses for the longest (>9 s), followed by IFT-A (~2 to 11 s) and IFT-dynein (~2.7 to 8 s) (*13*), as they bind to immobile assembling trains. In contrast, BBSomes only pause briefly (~0.4 to 0.7 s), similar to what we have observed before for kinesin-II (*13*), indicating that BBSomes associate to anterograde IFT trains that are completely assembled or at a relatively late stage of assembly. In line with this, BBSome docking locations are closer the TZ than those of IFT-A and IFT-B (Fig. 6, A to C). Our analysis reveals that assembling and fully assembled IFT trains have different affinities for several IFT components. Structural differences between assembling and moving trains have been reported (*12*), and one hypothesis suggests involvement of an unknown exogenous factor that assists train polymerization while also locking the train in an arrested conformation (*58*). Last, single-molecule localization maps in the proximal part of the cilium reveal that IFT-B is located closer to the axoneme than IFT-A and BBSomes, which are directed toward and might associate with the ciliary membrane. This observation complements structural studies of anterograde IFT trains (*9*, *58*, *59*) and highlights the role of IFT-A in mediating entry of signaling receptors into cilia (*60*–*62*). While the BBSome does not appear to play a role in ciliary entry of membrane proteins, it has been suggested to be involved in anterograde IFT of membrane proteins (*63*), which would explain its close proximity to the ciliary membrane.

In summary, with our single-molecule imaging approach, we have provided a dynamic view on how different ciliary components reach and organize at the PCMC, and associate with anterograde IFT trains to enter the cilium in wild-type *C. elegans*. In the future, our approach can be applied to investigate the nature of IFT trains in mutants with disrupted IFT and anterograde train assembly, providing insights into the functional roles of key components and regulators, such as the BBSome (*23*, *25*), TZ proteins (*13*, *35*), and regulatory proteins (*49*, *64*, *65*).

## METHODS

### *C. elegans* strains

The worm strains used in this study are listed in table S1. The strains used in this study have been generated using Mos-1–mediated single-copy insertion (*66*) and CRISPR-Cas9 genome editing (*67*). Maintenance was performed using standard *C. elegans* techniques (*68*), on nematode growth medium (NGM) plates, seeded with HB101 *Escherichia coli.*

### Fluorescence microscopy

Images were acquired using a custom-built laser-illuminated wide-field fluorescence microscope, as described previously (*69*). Briefly, optical imaging was performed using an inverted microscope body (Nikon Ti E) with a 100× oil immersion objective (Nikon, CFI Apo TIRF 100×, numerical aperture: 1.49) in combination with an EMCDD camera (Andor, iXon 897) controlled using MicroManager software (v1.4). A total of 491- and 561-nm diode-pumped solid state lasers (Cobolt Calypso and Cobolt Jive, 50 mW) were used for laser illumination. Laser power was adjusted using an acousto-optic tuneable filter (AOTF, AA Optoelectronics). For performing SWIM (*26*), the beam diameter was changed using an iris diaphragm (Thorlabs, SM1D12, ø 0.8 to 12 mm) mounted between the rotating diffuser and the epi lens, at a distance equal to the focal length of the latter. The full-beam width in the sample was ~30 μm (2σ of the Gaussian width). The aperture size of the diaphragm was adjusted manually to change the width of the beam, with a minimum beam width of ~7 μm at the sample, when the diaphragm is closed to a minimum diameter of 0.8 mm. Fluorescence light was separated from the excitation light using a dichroic mirror (ZT 405/488/561; Chroma) and emission filters (525/50 and 630/92 for collecting fluorescence excited by 491 nm and 561 nm, respectively; Chroma).

For imaging live *C. elegans*, young adult hermaphrodite worms, having PHA/PHB dendrites ~50 μm long, were sedated in 5 mM levamisole in M9, sandwiched between an agarose pad (2% agarose in M9) and a coverslip and mounted on a microscope (*69*). To perform SWIM, a small excitation window (width ranging between 7 and 15 μm) was used to illuminate fluorescent molecules (IFT proteins labeled with eGFP, mCherry, or wrmScarlet) in a small region of PHA/PHB neurons, to image protein dynamics in either the cilia or the dendrites (see Supplementary Movies).

Samples were typically imaged at 5.3× preamplifier gain and 800 electron-multiplying (EM) gain with 10 MHz analog-to-digital converter (ADC) readout, and pixel size of acquired images was 80 nm by 80 nm. To image ensemble dynamics and obtain average fluorescence-intensity profiles, a cilia pair was imaged with a low-intensity 491- or 561-nm beam (~0.1 mW/mm^2^ in the center of the beam) for 100 frames at 6.6 fps. To perform single-molecule imaging, the sample was illuminated with a high-intensity 491- or 561-nm beam (~10 mW/mm^2^ in the center of the beam). High-intensity illumination bleaches almost the whole pool of fluorescing molecules in the illuminated region of the sample, allowing visualization of fresh, not-yet-bleached single molecules entering the small illuminated region. The sample was imaged for 10 to 45 min, and image acquisition rate ranged between 4 and 100 fps, depending on the motility feature being visualized (diffusion, static, or directed motion) and the fluorescence protein being imaged (eGFP, mCherry, or wrmScarlet). For imaging diffusion of IFT components, the framerates were as follows: BBSome (eGFP::BBS-2) 50 fps; IFT-B (OSM-6::eGFP) 50 to 100 fps; IFT-A (CHE-11::mCherry) ~30 fps; IFT-dynein (XBX-1::eGFP) 50 to 100 fps; kinesin-II (KAP-1::eGFP) 50 to 100 fps; OCR-2 (OCR-2::eGFP) 20 to 50 fps. Directed transport of IFT-B– and IFT-A–coated vesicles in the dendrites was imaged at 4 to 20 fps (typically at 6.6 fps). Dynamics of single particles at ciliary base was imaged at 6.6 fps for IFT-A and at 6.6 fps or 20 fps for BBSome and IFT-B.

For alternating dual-color imaging of eGFP (OSM-6::eGFP; IFT-B) and wrmScarlet (CHE-11::wrmScarlet; IFT-A), an Arduino-compatible board (Adafruit ItsyBitsy M4) was used to enable communication between the camera and the AOTF module such that we alternate between 491 and 561 nm laser lines every 75 ms, as described by Zhang *et al.* (*30*). The emitted light was separated by a two-way beam splitter, collecting signal emanating from eGFP and wrmScarlet on different regions of the same camera chip.

### Image analysis

#### Intensity profiles

First, we generated a time-averaged projection of 100 frames of the cilium pair imaged at low laser intensity (see example projections in Fig. 1, C to E, top, and corresponding movies in movie S1). An intensity profile along the length of a cilium was made by measuring the intensity along a manually drawn segmented line (typical linewidth 9 pixels) using ImageJ. The background intensity was measured by placing a line of the same dimensions next to the cilium, which was subsequently subtracted from the raw signal to obtain the background-corrected value. The peak at the ciliary base was manually selected as 0 μm to align the intensity profiles of cilia from several worms. Intensity was normalized to the value at peak intensity. Average cilium-intensity profiles were obtained by averaging the intensity profiles of multiple individual cilia with error provided by the SD.

#### Single-molecule tracking considerations

Single-molecule tracking was only performed on those worms that did not show micrometer-range movement during the entire acquisition time (ranging between 8 and 45 min). Movement of the worm or image drift in the nanometer range could not be accounted for and could have a minor impact on the numbers we acquire from our analysis. The localization precision of individual fluorescing molecules is likely in the range of 40 nm (2σ), as estimated for surface-bound eGFP in our experimental setup (*24*), although it is slightly higher for moving molecules due to motion blur (*70*).

#### Tracking and spline fitting

Single-particle events were tracked using a MATLAB-based software, FIESTA (version 1.6.0) (*71*). Tracks, corresponding to an event, contain information regarding time (*t*_*i*_), *x* and *y* coordinates (*x*_*i*_, *y*_*i*_) and distance moved (*d*_*i*_), for every frame *i*. Minimum track length was 12 frames. Erroneous tracks, primarily caused by two (or more) single-molecule events too close to discriminate, were also excluded from further analysis (though used for fitting the spline). While retrograde events were observed during imaging and reported about anecdotally for BBSome (Fig. 3C), they were not tracked and analyzed further in this study. The strategy used in SWIM selects for fresh, not-yet-bleached particles entering the cilium, and for imaging single-molecule retrograde events in a statistically robust manner, we would need to use a different imaging strategy.

#### Transformation to ciliary coordinates

A ciliary coordinate system was defined by interpolating a cubic spline on a segmented line drawn along the long axis of the imaged dendrite/cilium, visualized via the single-molecule localizations obtained from tracking (see fig. S4A). Tracks excluded from further analysis were also included in visualizing the ciliary structure. A reference point was picked at the base of the characteristic “bone-shaped” structure, at the ciliary base. All single-molecule localizations were transformed from *x*- and *y*- coordinates (*x*_*i*_, *y*_*i*_) to ciliary coordinates ($c_{∥\_i}$, $c_{⊥\_i}$), with $c_{∥}$ the distance from the reference point along the spline and $c_{⊥}$ the distance perpendicular to the spline ($c_{⊥}$).

Since the characteristic bone-shaped structure at the ciliary base was slightly shifted for the different IFT components, the reference points were adjusted such that the shape of the velocity profiles along the cilium length (Fig. 6D) and the shape of the localization distribution (Fig. 6E) were aligned.

#### Classification of directed transport and pausing

To obtain a quantitative measure for the directedness of the motion, we used an MSD-based approach to extract the anomalous exponent (α) from $\mathrm{MSD}\left(\tau \right)=2\Gamma \tau^{\alpha }$ (where Γ is the generalized transport coefficient and τ is the time lag) along the track, in the direction of motion. α is a measure of the directedness of the motion, α = 2 for purely directed motion, α = 1 for purely diffusive motion, and α < 1 for subdiffusion or pausing. For each data point ($c_{∥\_i}$), we calculate α in the direction parallel to the spline ($\alpha_{∥\_i}$), using a windowed MSD classifier approach, described by Danné *et al.* (*72*). α was calculated analytically using the following equation

$$\alpha =\langle \alpha \rangle =\frac{2}{W-1}\;\sum_{n=1}^{\left(W-1\right)/2}\text{log}\left[\frac{MSD\left(n∆t+∆t\right)}{MSD\left(n∆t\right)}\right]/\text{log}\left(\frac{n+1}{n}\right)$$

keeping a fixed window (*W* = 12 frames). Because of the size of the window, all tracks shorter than 12 frames were removed from the analysis. Data points with $\alpha_{∥\_i}>1.2$ are classified as directed, and $\alpha_{∥\_i}<1$ are classified as paused.

#### Diffusivity measurement

The CVE was used to estimate the diffusion coefficient of diffusive tracks along the longitudinal axis of the dendrite using the following equation: $\hat{D}=\frac{\bar{{\left(∆x_{i}\right)}^{2}}}{2∆t}+\frac{\bar{∆x_{i}∆x_{i+1}}}{∆t}$, where $∆x_{i}$ = $c_{∥\_i+1}-c_{∥\_i}$ and ∆*t* is the frame time. CVE is optimal for estimating diffusion coefficients from short, noisy trajectories of freely diffusing particles in an unbiased and regression-free manner (*27*).

#### Velocity measurement

Before calculating the point-to-point velocity, the tracks were smoothened by rolling frame averaging more than 10 consecutive time frames to reduce the contribution due to localization error (typically estimated to be between 10 and 40 nm, depending on the brightness of the tracked object). The point-to-point velocity at a given localization (*x*_*i*_, *y*_*i*_) was calculated using the following equation: $v_{i=}\left(d_{i+1}-d_{i-1}\right)/\left(t_{i+1}-t_{i-1}\right)$. Only moving data points (see details of classification method below) are displayed in the Figs. 5F and 6D, where we calculated the average velocity and error of the velocity data at bins of 100 nm along the long axis of the cilia/dendrite, using bootstrapping.

#### Time-lag between vesicles in dendrite

The time-lag between subsequent vesicles moving across the dendrite, or arriving at the PCMC, was extracted by manually selecting events on a kymograph. The characteristic time lag (τ) was obtained by least-squares fitting of the cumulative distribution function (LSF-CDF), where the time lag distribution was used to generate a cumulative probability distribution that was fitted with the CDF: y=1−e−xτ. The average value and error were estimated using bootstrapping.

#### Measurement of vesicle intensity

The intensity of individual vesicles was estimated by manually drawing a box on the dendrite, with dimensions sufficient to capture all the light emitted by a single vesicle, positioned right at the edge of the illuminated spot, using ImageJ. Since the square was placed at the edge of the illumination spot, we minimized prior excitation and photobleaching that would decrease the measured intensity. The intensity of actively transported vesicles passing through the square was recorded. The background intensity was measured by placing a box of the same dimension outside the dendrite, and this was subsequently subtracted from the raw signal to obtain the background corrected value.

#### Numerical simulations of vesicle pools from which IFT-A and IFT-B coated vesicles are derived

Scenario 1: In the simulation, an exponential distribution of vesicles is generated with a characteristic time lag *t*_comb_ (*N* = 2500). Each vesicle is then randomly assigned as coated with either IFT-B only, IFT-A only, or both IFT-A and IFT-B, according to the proportions observed experimentally (Fig. 2L). Using these assignments, time lag distributions for IFT-A and IFT-B are obtained. The characteristic time lags, *t*_IFT-A_ and *t*_IFT-B,_ are then determined by LSF-CDF. *t*_comb_ is scanned from 4 to 8 s (step size, 0.1 s), and *t*_IFT-A_ and *t*_IFT-B_ are plotted with respect to each other (fig. S3B).

Scenario 2: In the simulation, two exponential time lag distributions are generated: *t*_IFT-B_only_, representing vesicles coated only with IFT-B, and *t*_both_, corresponding to vesicles coated with both IFT-A and IFT-B. Time lag between the vesicles from both these distributions is recorded until the cumulative time between selected vesicles reaches 10,000 s. The two distributions are then combined to create an overall distribution of vesicles coated with IFT-B. From this resultant distribution, the fraction of vesicles coated only with IFT-B and the characteristic time lag, *t*_IFT-B_, are determined. In fig. S3D, the fraction of vesicles coated only with IFT-B and *t*_IFT-B_ are plotted within the experimentally observed range, scanning *t*_IFT-B_only_ from 22 to 32 s (step size 0.1 s) and *t*_both_ from 6.5 to 8.5 s (step size 0.1 s).

#### Numerical simulations of 1D projection of 2D single-molecule localizations

Distribution of 2D localizations (*y*_*i*_, *z*_*i*_) along a transverse cross section of the modeled hollow cylinder representing the axoneme (scheme illustrated in fig. S3B), was numerically simulated (*N* = 100000), with *r*_*ax*_ = 150 nm providing the width of the cylinder, *r*_*db*_ = 25 nm providing the thickness of the ring, and localization precision estimated to be 40 nm (2σ for normal distribution). Coordinates of individual localizations were obtained as follows

$$y_{i}=r_{\mathrm{ax}}\times \text{cos}\theta_{\mathrm{ax}}+r_{\mathrm{db}}\times \text{cos}\theta_{\mathrm{db}}+{∆}_{i}$$

$$z_{i}=r_{\mathrm{ax}}\times \text{sin}\theta_{\mathrm{ax}}+r_{\mathrm{db}}\times \text{sin}\theta_{\mathrm{db}}+{∆}_{i}$$

where θ_*ax*_ and θ_*db*_ was randomly assigned to the localization and ${∆}_{i}$ (localization precision) was randomly picked from a normal distribution with width of 40 nm (2σ). The distribution of $y_{i}$, a bimodal distribution centered at 0, was fitted with a kernel density estimation, which provided the 1D projection distribution of localizations along the transverse cross section of a hollow cylinder. For simulating 2D localizations along the PCMC, we assumed a cylinder of width $r_{\text{PCMC}}$ = 280 nm (replacing $r_{\mathrm{ax}}$ in the above equations), negligible thickness ($r_{\mathrm{db}}$ = 0 nm) and ${∆}_{i}$ = 100 nm.

#### Filtering events at ciliary base

Tracks at the PCMC and the cilium were classified into vesicle events, stuck events, and entry events as follows: (i) Entry events: $c_{∥\_1}\geq -0.3\;\mu m$, $c_{∥\_\mathrm{end}}-c_{∥\_1}\geq 0.3\;\mu m$, and $\text{max}\left(c_{∥\_i}\right)>0.3\;\mu m$; (ii) PCMC events: $c_{∥\_1}\leq -0.5\;\mu m$ and $c_{∥\_\mathrm{end}}\leq 0.6\;\mu m$; and (iii) Stuck events: remaining events, where $c_{∥\_1}$ and $c_{∥\_\mathrm{end}}$ are the distance along the spline for the first and last data point of a given track. For BBSome tracks, each event that was not an entry event was classified as a stuck event.

#### Measurement of docking location

Docking location of a single-molecule entry track was defined as the averaged coordinates ($c_{∥}$, $c_{⊥}$) of the first two frames.

#### Measured pause time

Measured pause time, $t_{p\_m}$, of a single-molecule entry track was defined as the time taken (interpolated) to move the first 100 nm. Tracks shorter than 300 nm were discarded from the distribution.

#### Bleach time fit

The characteristic bleach time $t_{\text{bleach}}$ for a given fluorescently labeled protein in the cilia was obtained from the exponential fit ($e^{-x/t_{\text{bleach}}}$) to the decay in the fluorescence intensity (normalized) over time, as a result of bleaching induced by the high intensity laser exposure (as shown for OSM-6::eGFP in fig. S5B). The fluorescence intensity was measured on ImageJ by manually selecting a region containing fluorescent signal and a region next to it within the illuminated area, for background correction.

#### Numerical simulations of single-molecule trajectories

To determine the impact of bleaching on the measured pause time, entry events were numerically simulated (scheme illustrated in fig. S5A), similar to a previous study (*13*). Simulated tracks were allowed to dock at a given location along a 1D cilia lattice, with the docking location randomly picked from the distribution of docking locations obtained experimentally. Each track was assigned a bleach time ($t_{b}$) and a “real” pause time ($t_{p}$) randomly picked from exponential distributions with characteristic time $t_{\text{bleach}}$ (obtained from experiments) and $t_{p\_\text{real}}$ (free parameter), respectively. The location of the simulated event was updated every ∆t (with ∆t = 150 ms, as in most experiments), and the localization precision was estimated to be 40 nm (SD, 2σ). At frame I, while $t_{i}<t_{b}$ or $D_{i}$ < 4 μm, the location of the event was updated as follows

$$D_{1}\left(0\right)={∆}_{i};\text{if}\;i=1$$

$$D_{i}\left(t_{i}\right)=D_{i-1}\left(t_{i}-∆t\right)\pm {∆}_{i};\text{if}\;t_{i}<t_{p}$$

or

$$D_{i}\left(t_{i}\right)=D_{i-1}\left(t_{i}-∆t\right)+v_{i}\times ∆t\pm {∆}_{i};\text{if}\;t_{i}>t_{p}$$

where, $v_{i}=v_{\mathrm{avg}\_D_{i-1}}+∆v_{\mathrm{std}\_D_{i-1}}$ ($v_{\mathrm{avg}}$ is the location-dependent velocity at $D_{i-1}$ obtained from experiments; $∆v_{\mathrm{std}}$ is a randomly picked value from a normal distribution with width being the error in velocity at $D_{i-1}$ obtained from experiments), and ${∆}_{i}$ is the localization precision, randomly picked from a normal distribution with width 40 nm (2σ). For each simulation condition, with number of events *N*, the real pause time, $t_{p\_\text{real}}$, is varied, and the measured pause time, $t_{p\_m}$, is recorded for tracks longer than 300 nm, as described for experimental data.

#### Estimating the shape of the cilium from single-molecule localizations

Moving localizations of entry events were used to estimate the shape of the distribution of an IFT component along the cilia. The absolute distance perpendicular to the cilia spline was sampled every 1000 data points along the long axis of the cilia (shifting every 300 nm for the next sample) from the single-molecule localization map of a given imaged species. The 80 percentile value obtained from the CDF of the sampled distribution provided the width of the cilium at the location along the cilia spline where the distribution was sampled. We chose the 80 percentile value of the CDF since this is approximately where the distribution of absolute distance perpendicular to the cilia spline peaks for sampled BBSome localizations. The 70 to 90 percentile range of the CDF was displayed to represent the error in the width.

#### Estimating average value and error for distributions

We used a bootstrapping method to calculate the parameters of a distribution. We randomly selected *N* measurements from the distribution (with replacement) and calculated the median of the resampled group. We repeated this process 1000 times, creating a bootstrapping distribution of medians. We then calculated the mean (μ) and SD (σ) of this distribution and used these values to estimate the parameter and its error. Here, all values and errors are presented as μ ± 3σ.

#### Sample statistics

Single-molecule imaging at the dendrite and ciliary base of each *C. elegans* strain were repeated in at least two independent imaging sessions. The overview of the sample sizes, such as the number of organisms, cilia number, tracked events, and tracked localizations is provided in tables S2 and S3. Further, sample sizes are also mentioned in figures and figure captions.

#### Information on plots and figures

Kymographs were generated using the Multi Kymograph plug-in of Fiji/ImageJ. All the data were analyzed and plotted using custom written scripts on MATLAB (The Math Works, Inc., R2021a).

## Supplementary Materials

### The PDF file includes:

Supplemental text associated with fig. S3 Figs. S1 to S6 Tables S1 to S3 Legends for movies S1 to S7 References
