# Supplementary material for: Delivery of intraflagellar transport proteins to the ciliary base and assembly into trains
Source: Sci Adv. 2025 Apr 4;11(14):eadr1716. doi: 10.1126/sciadv.adr1716 (PMC11970479; doi:10.1126/sciadv.adr1716)
Supplement: Supplementary file 1 — Supplemental text associated with fig. S3 Figs. S1 to S6 Tables S1 to S3 Legends for movies S1 to S7 References [file sciadv.adr1716_sm.pdf]

Science Advances
AAAS

Supplementary Materials for

Delivery of intraflagellar transport proteins to the ciliary base and assembly into trains

Aniruddha Mitra *et al.*

Corresponding author: Aniruddha Mitra, a.mitra@uu.nl; Erwin J. G. Peterman, e.j.g.peterman@vu.nl

Sci. Adv. **11**, eadr1716 (2025)
DOI: 10.1126/sciadv.adr1716

The PDF file includes:

Supplemental text associated with fig. S3
Figs. S1 to S6
Tables S1 to S3
Legends for movies S1 to S7
References

Other Supplementary Material for this manuscript includes the following:

Movies S1 to S7

Supplemental text associated with Figure S3

Our analysis of directed dynamics of IFT-A and IFT-B coated vesicles in the dendrites of PHA/PHB neurons reveal a complex picture. Firstly, we find that IFT-A and IFT-B coated vesicles are moving across the dendrites at different rates – the characteristic time between subsequent IFT-B vesicles is 5.8 ± 0.5 s and between subsequent IFT-A vesicles is 8.9 ± 1 s (Figure 2I) – which suggests that they originate from different vesicle pools. However, dual-colour imaging shows that there is a significant, overlapping pool with 67% of the vesicles carrying both IFT-A and IFT-B subcomplexes, while 26.5% of vesicles carry only IFT-B and 6.5% of the vesicles carry only IFT-A (Figure 2K). Here we perform numerical simulations to explore whether the contrasting dynamics of IFT-A- and IFT-B-coated vesicles —despite a significant proportion of vesicles carrying both IFT-A and IFT-B— can be explained by either these vesicles originating from a single exponential pool or from multiple sub-pools that are differentially sorted at the soma. We consider two different scenarios, which are as follows:

Scenario 1: Vesicles carrying only IFT-A, only IFT-B and both IFT subcomplexes are all derived from the same exponential pool with a characteristic time lag t_{comb} between vesicles (Figure S3A). The different sub-pools of vesicles are in the ratio 6.5:26.5:67, as observed in our experiments (Figure 2L). From a simulation of an exponential pool with time lag t_{comb} (N = 2500 vesicles), we obtain the characteristic time lag corresponding to all IFT-A associated vesicles ($t_{IFT\text{-}A}$) and all IFT-B associated vesicles ($t_{IFT\text{-}B}$). We scan t_{comb} over 4 to 8 s (step size 0.1 s) and find that $t_{IFT\text{-}A}$ in the range 8.9 ± 1.0 s (value in experiments; Figure 2J) predicts a $t_{IFT\text{-}B}$ in the range 6.2 – 7.8 s while $t_{IFT\text{-}B}$ in the range 5.8 ± 0.5 s (value in experiments; Figure 2J) predicts a $t_{IFT\text{-}A}$ in the range 6.7 – 8.1 s (Figure S3B). Since the predicted values do not entirely match the values that we obtain from experiments it is very likely that this is not the scenario within the PHA-PHB neurons.

Scenario 2: IFT-B only vesicles and vesicles containing both IFT-A and IFT-B are derived from different pools, each characterized by distinct exponential time constants ($t_{IFT\text{-}B\_only}$ and t_{both}, respectively; Figure S3C). From a simulation, for a given $t_{IFT\text{-}B\_only}$ and t_{comb}, we obtain values for $t_{IFT\text{-}B}$ and the fraction of IFT-B only vesicles. In our experiments, we find that the fraction of IFT-B only vesicles is ~28% (Fraction[IFT-B only]/(Fraction[IFT-B only] + Fraction[both IFT-A and IFT-B]))and $t_{IFT\text{-}B}$ is 5.8 ± 0.5 s. We perform simulations, scanning over different combinations of $t_{IFT\text{-}B\_only}$ and t_{comb}, and find that a $t_{IFT\text{-}B\_only}$ in the range of 24–30 s and a t_{both} in the range of 7–8 s yield results consistent with our experimental observations of $t_{IFT\text{-}B}$ and the fraction of IFT-B-only vesicles (Figure S3D). This scenario provides a plausible explanation for the observed experimental results.

Thus, if we consider that IFT-B coated vesicles are derived from two different pools of vesicles we can already provide an explanation for the experimental results. It is likely that the actual scenario is far more complex, with multiple overlapping vesicle pools at the soma, each containing varied compositions of IFT-A and IFT-B subcomplexes.

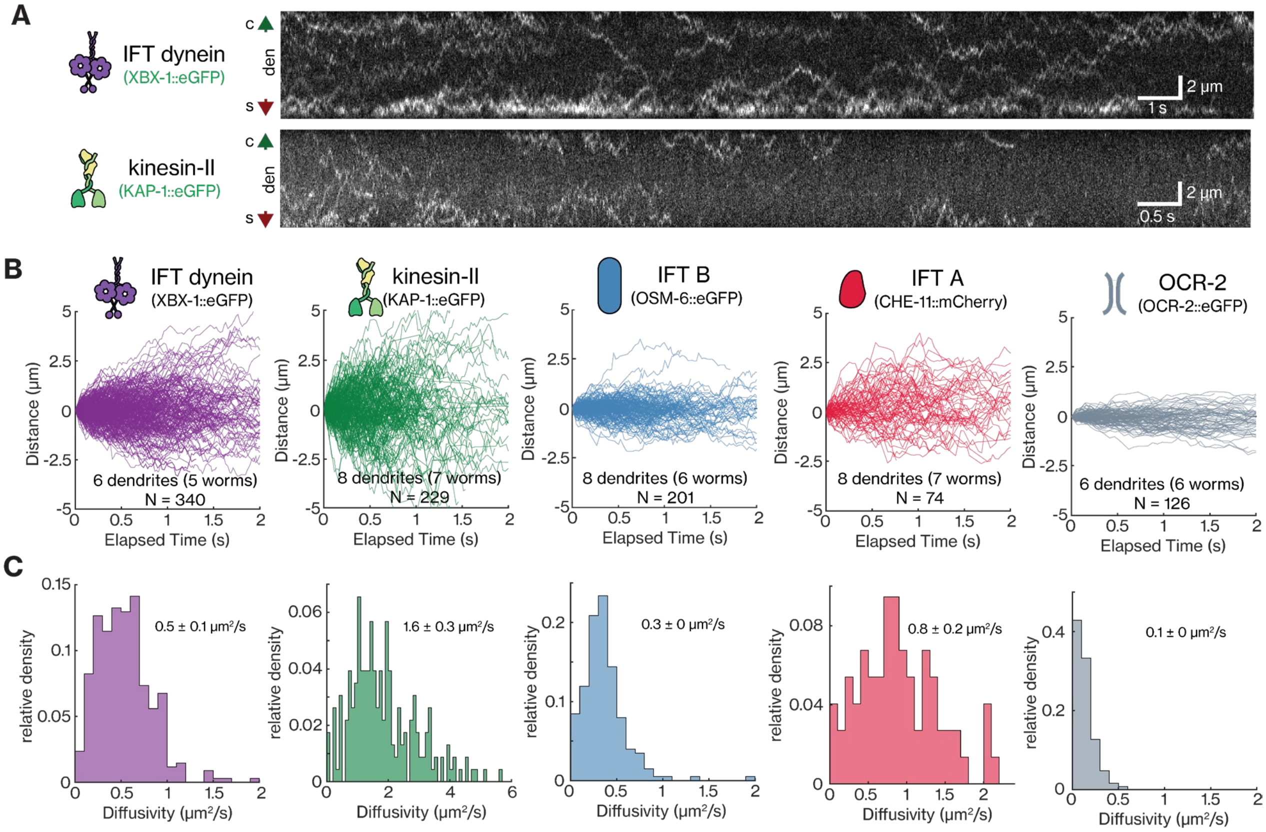

Figure S1: Analysis of tracked diffusive events of different IFT components diffusing in the dendrites of PHA/PHB neurons. (A) Representative kymograph of IFT dynein (XBX-1::eGFP; light intermediate chain subunit of IFT-dynein; top) and kinesin-II (KAP-1::eGFP; non-motor subunit of heterotrimeric kinesin-2; bottom) displays that single IFT dynein and kinesin-II motors diffuse across the dendrite (see Movie S2). Green and red arrows indicate the cilium and soma direction, respectively. **(B-C)** Distance-time plots (B) and histogram of the diffusivity of individual tracks (C) for different IFT components: IFT dynein: 340 tracks in 6 dendrites, average diffusivity $0.5 \pm 0.1\ \mu m^2/s$; kinesin-II: 229 tracks in 8 dendrites, average diffusivity $1.6 \pm 0.3\ \mu m^2/s$; IFT-B (OSM-6::eGFP): 201 tracks in 8 dendrites, average diffusivity 0.3 ± 0 $\mu m^2/s$; IFT-A (CHE-11::mCherry): 74 tracks in 8 dendrites, average diffusivity $0.8 \pm 0.2\ \mu m^2/s$; OCR-2 associated vesicles (OCR-2::eGFP): 126 tracks in 6 dendrites, average diffusivity 0.1 ± 0 $^2/s$ (data from Mitra et al. (*26*)). Average values and errors are estimated using bootstrapping.

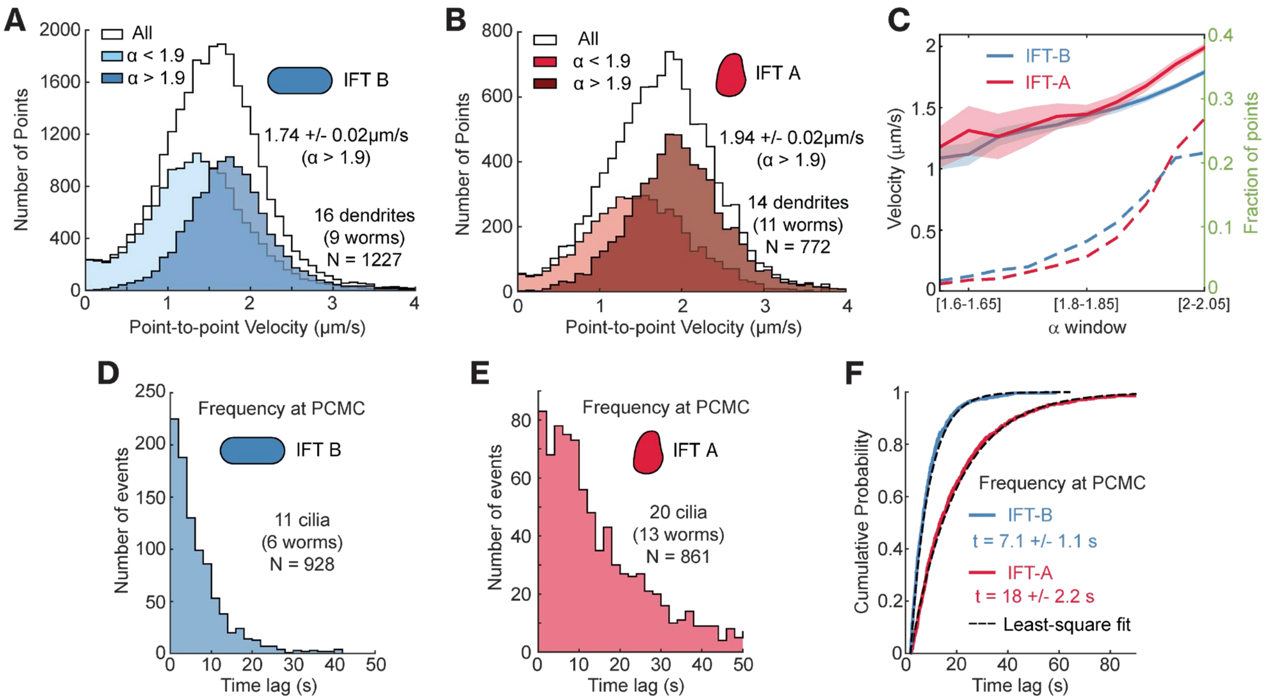

Figure S2: Analysis of the motion of directed vesicles coated with IFT-B and IFT-B moving across dendrites from soma to cilia and at the PCMC. (A) Histograms of point-to-point velocities obtained from 1277 tracks of IFT-B (OSM-6) coated vesicles from 16 dendrites. Average velocity obtained from all data points is 1.54 ± 0.01 μm/s (N = 26765; grey). For data points with $\alpha < 1.9$, average velocity is 1.33 ± 0.02 μm/s (N = 15125; light blue), while, for data points with $\alpha > 1.9$, it is 1.74 ± 0.02 μm/s (N = 11640; blue). **(B)** Histograms of point-to-point velocities obtained from 772 tracks of IFT-A (CHE-11) packets from 14 dendrites. Average velocity obtained from all data points is 1.76 ± 0.02 μm/s (N = 10910; grey). For data points with $\alpha < 1.9$, average velocity is 1.45 ± 0.04 μm/s (N = 4738; red), while, for data points with $\alpha > 1.95$, it is 1.94 ± 0.02 μm/s (N = 6172; brown). **(C)** Average velocity of data points binned over α windows with bin width = 0.05, moving from 1.6 to 2.05. Number of data points in each bin is indicated by dotted line (right y-axis). Data corresponding to IFT-A (red) and IFT-B (blue) coated vesicles, respectively. Number of data points in each bin is indicated by dotted line (right y-axis). **(D)** Histogram of time lags between subsequent IFT-B coated vesicles arriving at the PCMC (11 neurons; N = 928). **(E)** Histogram of time lags between subsequent IFT-A coated vesicles arriving at the PCMC (20 neurons; N = 861). **(F)** Time-lag distribution of IFT-B (blue) and IFT-A (red) coated vesicles arriving at the PCMC plotted as a cumulative distribution function, overlayed with the least square single exponential fit to the function. The characteristic time between subsequent IFT-B vesicles is 7.1 ± 1.1 s and between subsequent IFT-A vesicles is 18 ± 2.2 s. Average value and error are estimated using bootstrapping (see Methods).

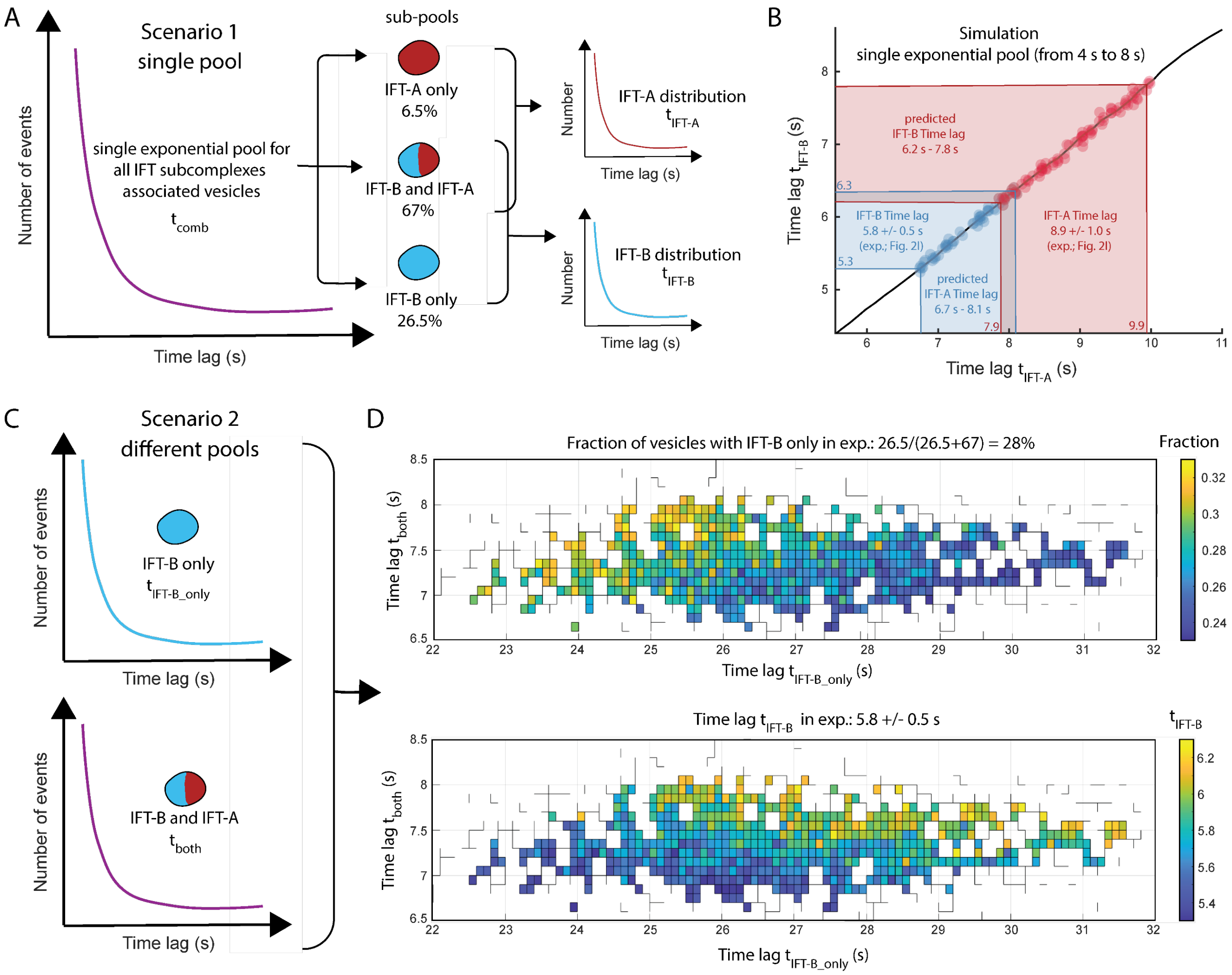

Figure S3: Numerical simulations considering either a single pool or multiple pools of vesicles coated with IFT-A and IFT-B subcomplexes. (A) Vesicles coated with IFT-A, IFT-B and both are all drawn from a single exponential pool with a characteristic time lag t_{comb}. From this we can calculate the characteristic time lag for all IFT-A (t_{IFT-A}) and IFT-B (t_{IFT-B}) coated vesicles, with the information from experiments (Figure 2K) that 6.5% of vesicles contains only IFT-A, 26.5% contains only IFT-B and 67% contains both IFT-A sand IFT-B. **(B)** Plot of t_{IFT-B} as a function of t_{IFT-A}, sampling t_{comb} from 4 s to 8 s (step size 0.1 s; number of vesicles N = 2500). t_{IFT-A} in the range 8.9 ± 1.0 s (from experiments; Figure 2I) predicts a t_{IFT-B} in the range 6.2 – 7.8 s (red shaded region) while t_{IFT-B} in the range 5.8 ± 0.5 s (from experiments; Figure 2I) predicts a t_{IFT-A} in the range 6.7 – 8.1 s (blue shaded region). **(C)** Scenario 2: Vesicles coated with IFT-B only and vesicles coated with both IFT-B and IFT-A are derived from different exponential pools, with characteristic time lag t_{IFT-B_only} and t_{both}, respectively. **(D)** Each simulation (number of vesicles N = 2500) with a given t_{IFT-B_only} (sampled from 22 s to 32 s with step size 0.1 s) and t_{both} (sampled from 6.5 s to 8.5 s with step size 0.1 s) yields a value for t_{IFT-B} and fraction of vesicles carrying only IFT-B. Here we plot t_{both} as a function of t_{IFT-B_only} with the color indicating the value of fraction of vesicles carrying only IFT-B (upper panel) in the range 0.23 - 0.33 (obtained from experiments) and t_{IFT-B} (lower panel) in the range 5.3 s – 6.3 s.

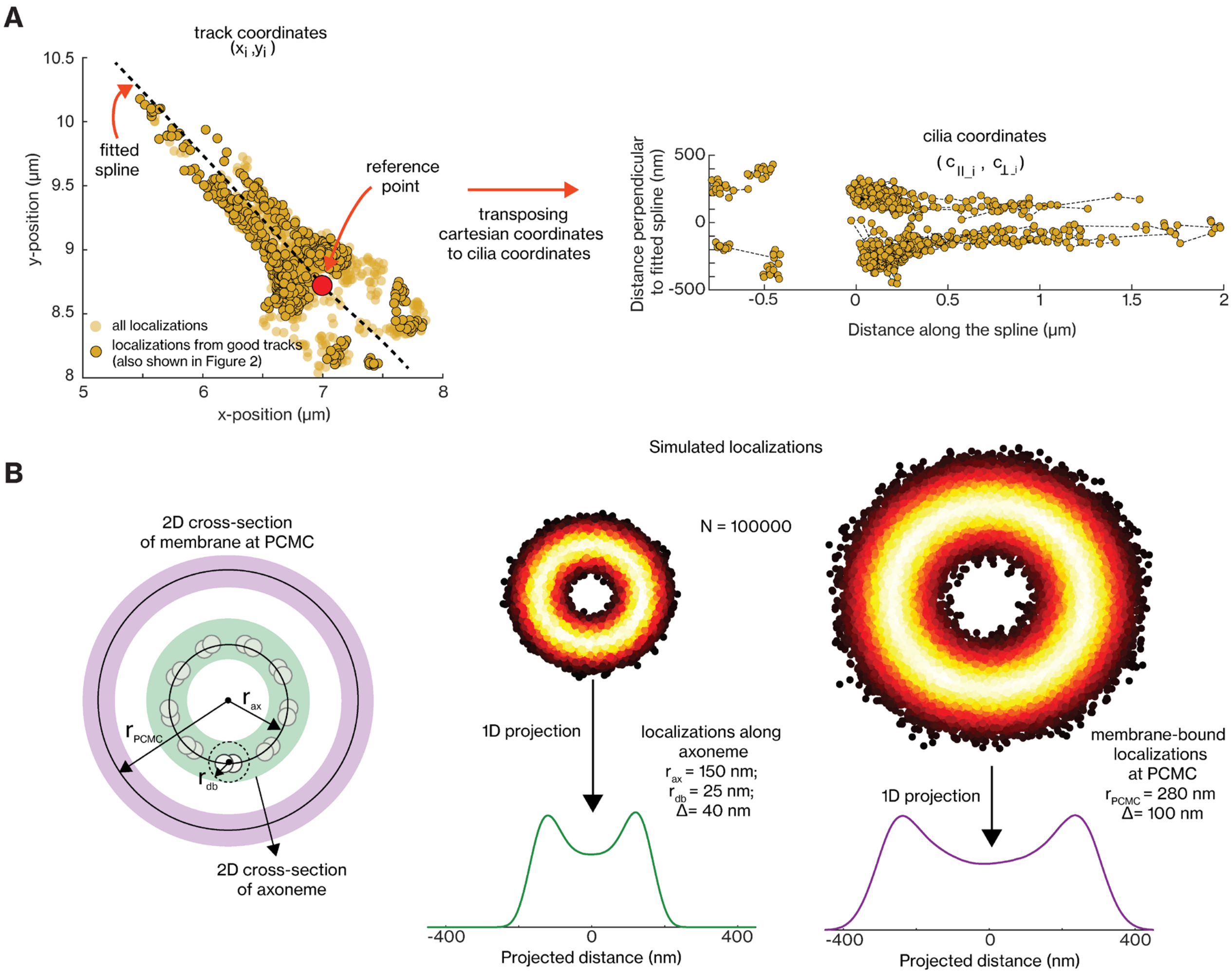

Figure S4: (A) Transposing cartesian coordinates of single-molecule tracks to general cilia coordinates. Top: x-y coordinates of all tracked kinesin-II entry events (846 single-molecule localizations from 18 tracks) in an imaged cilium. The single-molecule localizations provide the structure near the ciliary base, making it possible to draw a spline (dotted black line) roughly along the longitudinal axis of the cilium and select a reference point at the ciliary base. The cartesian coordinates of each single-molecule localization (x_i, y_i) can be transposed to distance perpendicular to the spline $(c_{\perp i})$ and distance from the reference point $(c_{\| i})$, referred to as cilia coordinates. Bottom: Single-molecule localizations replotted in cilia coordinates. **(B)** 1D projection simulations to explain the bimodal distribution (centred around 0 μm) observed for single-molecule localizations in the direction perpendicular to the fitted spline. Left: Illustration of the transverse section of the hollow axoneme (radius r_{ax}; shaded in green) comprising of 9 microtubule doublets, with BBSome complexes moving along individual doublets within the radius (radius r_{db}) and the transverse section occupied by membrane bound BBSome complexes at PCMC (radius r_{PCMC}; shaded in purple). Right: 1D projection of the 2D cross-sectional geometry illustrated in the left. Parameters for axonemal cross-section: N = 10000; r_{ax} = 150 nm, r_{db} = 25nm and localization error Δ= 40nm. Parameters for membrane cross-section: N = 10000; r_{ax} = 280 nm and error Δ_{PCMC}= 100nm. Δ_{PCMC} accounts for localization error and variability in the radius of the PCMC between worms.

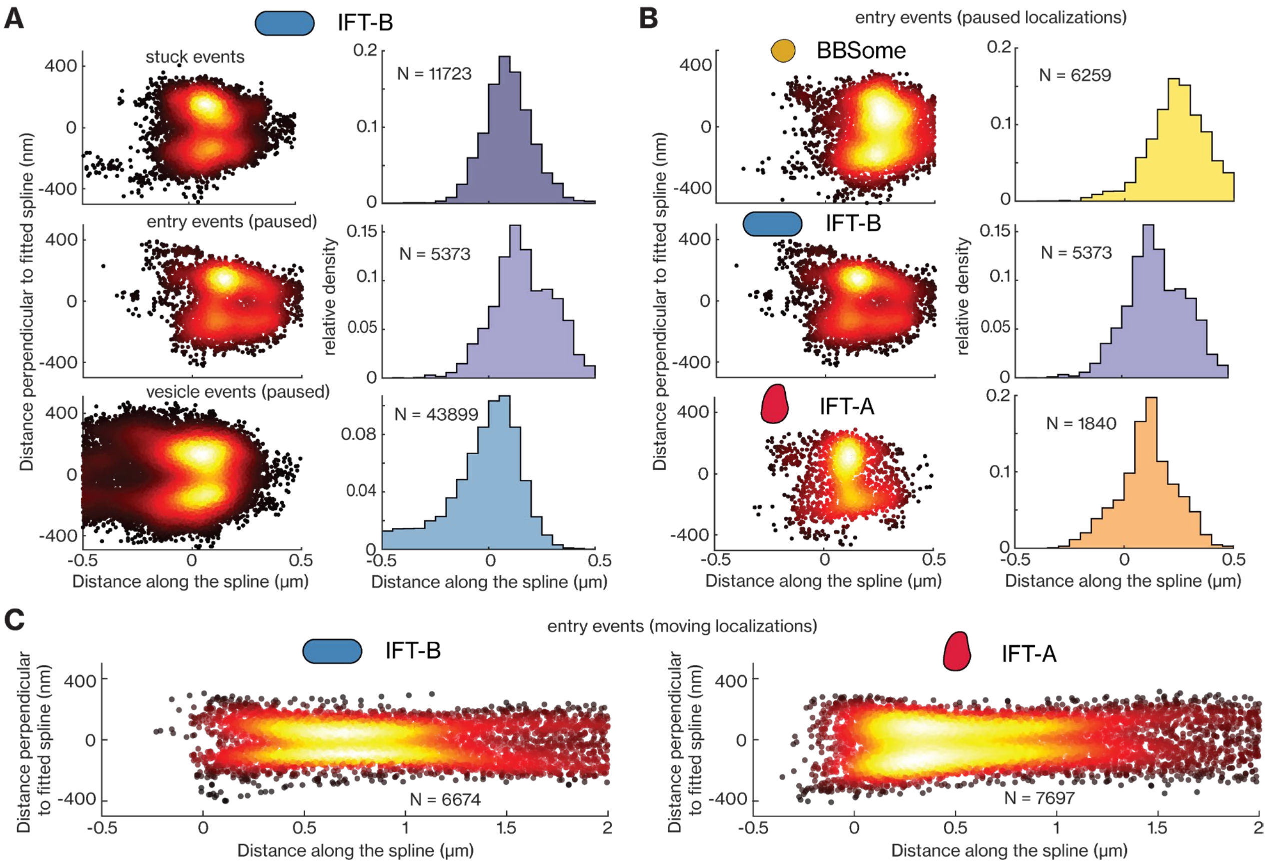

Figure S5: Single-particle localizations of IFT-B and IFT-A in the PCMC and ciliary base. (A) Super-resolution map of paused single-particle localizations (left) and distribution of the localizations along the longitudinal axis of the cilia (right), obtained from tracks of IFT-B. Top: paused localizations from stuck events (11723 localizations from 245 tracks); middle: paused localizations from entry events (5373 localizations from 154 tracks); bottom: paused localizations from vesicle events (43899 localizations from 1549 tracks). **(B)** Super-resolution map of paused localizations (left) and distribution of the localizations along the longitudinal axis of the cilia (right) obtained from entry events of BBSome (top; 6259 localizations), IFT-B (middle; 5373 localizations) and IFT-A (bottom; 1840 localizations). **(C)** Super-resolution map of moving localizations of entry events for IFT-B (left; 6674 localizations) and IFT-A (right; 7697 localizations).

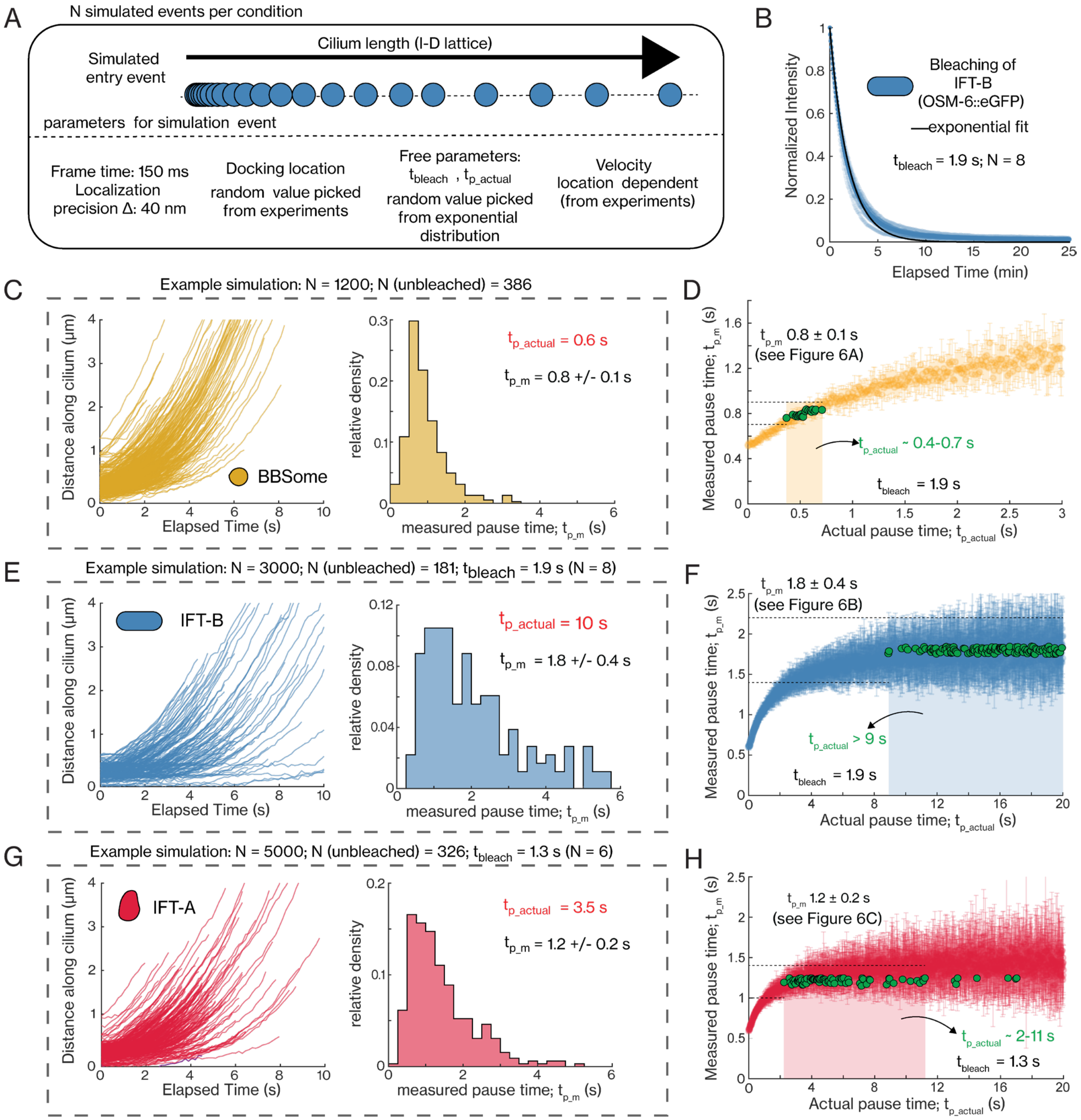

Figure S6: Numerical simulations to estimate actual pause time of single-molecule tracks entering cilia. (A) Scheme of the numerical simulation, as also performed previously (*13*). Each simulated molecule is designated a bleach time (t_b) and "actual" pause time (t_p), randomly picked from exponential distributions with rate parameters t_{bleach} (estimated from experiments) and t_{p_actual} (free parameter), respectively. The molecule docks along a 1D cilium lattice, with the docking location randomly selected from experimentally measure docking locations. After every time interval, the molecule either stays in the same location (elapsed time $t < t_b \,\&\, t_p$), moves forward ($t < t_b \,\&\, t > t_p$) with a location dependent velocity (obtained from experiments) or beaches ($t \geq t_b$; end of event). N is the number of events for a given condition, frame time is 150 ms and the localization precision is 40 nm (2σ). **(B)** Exponential decay of the IFT-B (OSM-6::eGFP) intensity over time, upon exposure to high intensity 491 nm laser (number of cilia N = 8). The exponential fit (black line) provides a characteristic t_{bleach} = 1.9 s. **(C)** Distance-time plots of simulated BBSome entry events (N = 1200, N[unbleached] = 386) and histogram of measured

pause time (average pause time t_{p_m} = 0.8 $\pm$ 0.1 s). **(D)** Distribution of the measured pause times, t_{p_m}, with respect to actual pause times, t_{p_actual}, obtained from numerical simulations of BBSome entering cilia, assuming a characteristic bleach time (t_{bleach}) of 1.9 s. Each point represents the average pause time (t_{p_m}) for a given simulated experiment. t_{p_actual} is in the range 0.4-0.7s for t_{p_m} 0.8 $\pm$ 0.1nm (experimentally obtained; Figure 6A) **(E)** Distance-time plots of simulated IFT-B entry events (N = 3000, N[unbleached] = 181) and histogram of measured pause time (average pause time t_{p_m} = 1.8 $\pm$ 0.4 s), for t_{p_actual} = 10s. **(F)** Distribution of the measured pause time, t_{p_m}, with respect to actual pause time, t_{p_actual}, for IFT-B, obtained from numerical simulations (using t_{bleach} = 1.9 s). t_{p_actual} is estimated to be > 9 s for t_{p_m} = 1.8 $\pm$ 0.4s (experimentally obtained; Figure 6B). **(G)** Distance-time plots of simulated IFT-A entry events (N = 5000, N[unbleached] = 326) and histogram of measured pause time (average pause time t_{p_m} = 1.2 $\pm$ 0.2 s), for t_{p_actual} = 3.5 s. t_{bleach} is 1.3 s (N = 6). **(H)** Distribution of the measured pause time, t_{p_m}, with respect to actual pause time, t_{p_actual}, for IFT-A, obtained from numerical simulations (using t_{bleach} = 1.3 s). t_{p_actual} is estimated to be in the range 2-11 s for t_{p_m} = 1.2 $\pm$ 0.2s (experimentally obtained; Figure 6C). Average value and error are estimated using bootstrapping.

Strain	Genotype	Source	Short notation
PHX6589	*bbs-2(syb6589[eGFP::bbs-2]) IV*	This study (made by SunyBiotech)	BBSome (BBS-2)
EJP76	*vuaSi15 [pBP36; Posm-6::osm-6::eGFP; cb-unc-119(+)] I; unc-119(ed3) III; osm-6(p811) V*	Prevo et al., 2015 (*24*)	IFT-B (OSM-6)
EJP81	*vuaSi24 [pBP43; Pche-11::che-11::mCherry; cb-unc-119(+)] II; unc-119(ed3) III; che-11(tm3433) V*	Prevo et al., 2015 (*24*)	IFT-A (CHE-11)
EJP13	*kap-1(ok676) III; vuaSi1 [pBP20; Pkap-1::kap-1::eGFP; cb-unc-119(+)] IV*	Prevo et al., 2015 (*24*)	kinesin-II
EJP212	*vuaSi26 [Pxbx-1::xbx-1::EGFP; cb-unc119(+)] I; vuaSi2 [Posm-3::osm3::mCherry; cb-unc-119(+)] II; osm3(p802) IV; xbx-1(ok279) V*	Mijalkovic et al., 2017 (*73*)	IFT-dynein
PHX8141	*osm-6(syb7957[osm-6::eGFP)V,che-11(syb8141[che-11::wrmScarlet])V*	This study (made by SunyBiotech)	IFT-B and IFT-A

Table S1: *C. elegans* strains used in this study. Short notation is used throughout the main text and figures to increase readability.

Worm strain	motility state	# worms	# dendrite	# tracks	Diff. ($\mu m^2/s$)	Velocity ($\mu m/s$) (# localizations)	Freq. (s^{-1}) (# packets)
BBSome (BBS-2)	Diffusive	10	11	330	0.8 ± 0.1	-	-
IFT-B (OSM-6)	Diffusive	6	8	201	0.3 ± 0	-	-
	Directed	9	16	1227	-	All: 1.54 ± 0.01 (26765)<br>α > 1.9: 1.74 ± 0.02 (11640)<br>α < 1.9: 1.33 ± 0.2 (15125)	Dendrite: 5.8 ± 0.5 (2375)<br>PCMC: 7.1 ± 1.1 (928)
IFT-A (CHE-11)	Diffusive	7	8	74	0.8 ± 0.2	-	-
	Directed	11	14	772	-	All: 1.76 ± 0.02 (10910)<br>α > 1.9: 1.94 ± 0.02 (6172)<br>α < 1.9: 1.45 ± 0.3 (4738)	Dendrite: 8.9 ± 1 (1130)<br>PCMC: 18 ± 2.2 (861)
IFT dynein	Diffusive	5	6	340	0.5 ± 0.1	-	-
kinesin-II	Diffusive	7	8	229	1.6 ± 0.3	-	-
OCR-2	Diffusive	6	6	126	0.1 ± 0	-	-

Table S2: Data collected from tracking single-particle events of different IFT components in the dendrites of PHA/PHB neurons.

Worm strain	# worms	# cilia	# tracks	Moving loc. ($\alpha > 1.2$)	Paused loc. ($\alpha < 1$)	Docking Loc. (nm)	t_{p_m} (s)	t_{p_actual} (s)
BBS-2 (BBSome)	27	46	Stuck: 456	437	13623	-	-	-
			Entry: 367	11358	6517	185 ± 23	0.8 ± 0.1	0.4-0.7
OSM-6 (IFT-B)	16	30	PCMC:1549	42192	43899	-	-	-
			Entry: 154	6674	5373	98 ± 28	1.8 ± 0.4	> 9
			Stuck: 245	136	11723	-	-	-
CHE-11 (IFT-A)	20	37	PCMC: 727	11011	7729	-	-	-
			Entry: 297	7697	1840	71 ± 26	1.2 ± 0.2	2-11
			Stuck: 264	409	6443	-	-	-

Table S3: Data collected from tracking single molecule events of IFT train complexes in the PCMC and cilia of PHA/PHB neurons.

Movie Captions

Movie S1: Example movies of dynamics of BBSome (eGFP::BBS-2; left), IFT-B (OSM-6::eGFP; middle) and IFT-A (CHE-11:mCherry; right) in the PHA/PHB cilia of *C. elegans*, acquired at low laser intensity. Movies play at 3x real time. Time and scale bar indicated. Related to Figure 1.

Movie S2: (A-C) Example movies (bottom panel) of single-molecule diffusive dynamics of BBSome (eGFP::BBS-2; A), IFT dynein (XBX-1::eGFP; B) and kinesin-II (KAP-1::eGFP; C) in the dendrites of PHA/PHB neurons, imaged using SWIM. Top-panel show the maximum projection corresponding to the movies, where the location of the dendrite(s) is indicated. Movies play at real time. Time and scale bar indicated. Related to Figures 2A-2C; Figure S1.

Movie S3: (A-B) Example movies (bottom panel) of single-particle diffusive and directed dynamics of IFT-B (A) and IFT-A (B) in the dendrites of PHA/PHB neurons, imaged using SWIM. Top-panel show the maximum projection corresponding to the movies, where the location of the dendrite(s) is indicated. Movies play at 3x real time. Time and scale bar indicated. Related to Figures 2F-2I; Figure S2.

Movie S4: Example movie obtained from dual-colour imaging of IFT-B (OSM-6::eGFP) and IFT-A (CHE-11:wrmScarlet) in the dendrites of PHA/PHB neurons. Left: green channel, middle: red channel and right: merged. Movies play at 3x real time. Time and scale bar indicated. Related to Figures 2J-2K.

Movie S5: Example movie (right-panel) displaying single-particle dynamics of individual BBSome complexes at the PCMC and proximal part of cilia. Left-panel shows the maximum projection corresponding to the movie, where the location of the dendrites, cilia and ciliary base are indicated. Movies play at 3x real time. Time and scale bar indicated. Related to Figure 3.

Movie S6: (A) Example movie (right-panel) displaying single-particle dynamics of individual IFT-B complexes at the PCMC and proximal part of cilia. Left-panel shows the maximum projection corresponding to the movie, where the location of the dendrites, cilia and ciliary base are indicated. Movies play at 3x real time. **(B)** Sections of the movie in A displaying example IFT-B events. Left: Directed packets of IFT-B moving from the dendrite into the PCMC. Right: Ciliary entry event and a directed packet event releasing IFT-B complexes that diffuse. Movies play at real time. In all movies, time and scale bar indicated. Related to Figure 4A-4C.

Movie S7: (A) Example movie (right-panel) displaying single-particle dynamics of individual IFT-A complexes at the PCMC and proximal part of cilia. Left-panel shows the maximum projection corresponding to the movie, where the location of the dendrites, cilia and ciliary base are indicated. Movies play at 3x real time. **(B)** Sections of the movie in A displaying example IFT-A events. Left: Directed packets of IFT-A moving from the dendrite into the PCMC. Right: Ciliary entry events. Movies play at real time. In all movies, time and scale bar indicated. Related to Figures 4D-4F

REFERENCES AND NOTES

1. M. V. Nachury, D. U. Mick, Establishing and regulating the composition of cilia for signal transduction. *Nat. Rev. Mol. Cell Biol.* **20**, 389–405 (2019).

2. W. Mul, A. Mitra, E. J. G. Peterman, Mechanisms of regulation in intraflagellar transport. *Cells* **11**, 2737 (2022).

3. B. Prevo, J. M. Scholey, E. J. G. Peterman, Intraflagellar transport: Mechanisms of motor action, cooperation, and cargo delivery. *FEBS J.* **284**, 2905–2931 (2017).

4. F. R. Garcia-Gonzalo, J. F. Reiter, Open sesame: How transition fibers and the transition zone control ciliary composition. *Cold Spring Harb. Perspect. Biol.* **9**, a028134 (2017).

5. M. V. Nachury, E. S. Seeley, H. Jin, Trafficking to the ciliary membrane: How to get across the periciliary diffusion barrier? *Annu. Rev. Cell Dev. Biol.* **26**, 59–87 (2010).

6. D. K. Breslow, E. F. Koslover, F. Seydel, A. J. Spakowitz, M. V. Nachury, An in vitro assay for entry into cilia reveals unique properties of the soluble diffusion barrier. *J. Cell Biol.* **203**, 129–147 (2013).

7. H. L. Kee, J. F. Dishinger, T. L. Blasius, C.-J. Liu, B. Margolis, K. J. Verhey, A size-exclusion permeability barrier and nucleoporins characterize a ciliary pore complex that regulates transport into cilia. *Nat. Cell Biol.* **14**, 431–437 (2012).

8. M. A. Jordan, G. Pigino, The structural basis of intraflagellar transport at a glance. *J. Cell Sci.* **134**, jcs247163 (2021).

9. M. A. Jordan, D. R. Diener, L. Stepanek, G. Pigino, The cryo-EM structure of intraflagellar transport trains reveals how dynein is inactivated to ensure unidirectional anterograde movement in cilia. *Nat. Cell Biol.* **20**, 1250–1255 (2018).

10. J. V. K. Hibbard, N. Vazquez, R. Satija, J. B. Wallingford, Protein turnover dynamics suggest a diffusion-to-capture mechanism for peri-basal body recruitment and retention of intraflagellar transport proteins. *Mol. Biol. Cell* **32**, 1171–1180 (2021).

11. J. L. Wingfield, I. Mengoni, H. Bomberger, Y.-Y. Jiang, J. D. Walsh, J. M. Brown, T. Picariello, D. A. Cochran, B. Zhu, J. Pan, J. Eggenschwiler, J. Gaertig, G. B. Witman, P. Kner, K. Lechtreck, IFT trains in different stages of assembly queue at the ciliary base for consecutive release into the cilium. *eLife* **6**, e26609 (2017).

12. H. van den Hoek, N. Klena, M. A. Jordan, G. Alvarez Viar, R. D. Righetto, M. Schaffer, P. S. Erdmann, W. Wan, S. Geimer, J. M. Plitzko, W. Baumeister, G. Pigino, V. Hamel, P. Guichard, B. D. Engel, In situ architecture of the ciliary base reveals the stepwise assembly of intraflagellar transport trains. *Science* **377**, 543–548 (2022).

13. A. Mitra, E. Loseva, E. J. G. Peterman, IFT cargo and motors associate sequentially with IFT trains to enter cilia of *C. elegans*. *Nat. Commun.* **15**, 3456 (2024).

14. H. Yang, K. Huang, Dissecting the vesicular trafficking function of IFT subunits. *Front. Cell Dev. Biol.* **7**, 352 (2020).

15. C. T. Baldari, J. Rosenbaum, Intraflagellar transport: It's not just for cilia anymore. *Curr. Opin. Cell Biol.* **22**, 75–80 (2010).

16. G. Jekely, D. Arendt, Evolution of intraflagellar transport from coated vesicles and autogenous origin of the eukaryotic cilium. *Bioessays* **28**, 191–198 (2006).

17. T. J. P. van Dam, M. J. Townsend, M. Turk, A. Schlessinger, A. Sali, M. C. Field, M. A. Huynen, Evolution of modular intraflagellar transport from a coatomer-like progenitor. *Proc. Natl. Acad. Sci. U.S.A.* **110**, 6943–6948 (2013).

18. T. Sedmak, U. Wolfrum, Intraflagellar transport molecules in ciliary and nonciliary cells of the retina. *J. Cell Biol.* **189**, 171–186 (2010).

19. C. R. Wood, J. L. Rosenbaum, Proteins of the ciliary axoneme are found on cytoplasmic membrane vesicles during growth of cilia. *Curr. Biol.* **24**, 1114–1120 (2014).

20. C. R. Wood, Z. Wang, D. Diener, J. M. Zones, J. Rosenbaum, J. G. Umen, IFT proteins accumulate during cell division and localize to the cleavage furrow in Chlamydomonas. *PLOS ONE* **7**, e30729 (2012).

21. T. Quidwai, J. Wang, E. A. Hall, N. A. Petriman, W. Leng, P. Kiesel, J. N. Wells, L. C. Murphy, M. A. Keighren, J. A. Marsh, E. Lorentzen, G. Pigino, P. Mill, A WDR35-dependent coat protein complex transports ciliary membrane cargo vesicles to cilia. *eLife* **10**, e69786 (2021).

22. J. V. K. Hibbard, N. Vazquez, J. B. Wallingford, Cilia proteins getting to work—How do they commute from the cytoplasm to the base of cilia? *J. Cell Sci.* **135**, jcs259444 (2022).

23. Q. Wei, Y. Zhang, Y. Li, Q. Zhang, K. Ling, J. Hu, The BBSome controls IFT assembly and turnaround in cilia. *Nat. Cell Biol.* **14**, 950–957 (2012).

24. B. Prevo, P. Mangeol, F. Oswald, J. M. Scholey, E. J. G. Peterman, Functional differentiation of cooperating kinesin-2 motors orchestrates cargo import and transport in *C. elegans* cilia. *Nat. Cell Biol.* **17**, 1536–1545 (2015).

25. G. Ou, O. E. Blacque, J. J. Snow, M. R. Leroux, J. M. Scholey, Functional coordination of intraflagellar transport motors. *Nature* **436**, 583–587 (2005).

26. A. Mitra, E. Loseva, G. H. Haasnoot, E. J. G. Peterman, A small excitation window allows long-duration single-molecule imaging, with reduced background autofluorescence, in *C. elegans* neurons. *Opt. Commun.* **545**, 129700 (2023).

27. C. L. Vestergaard, P. C. Blainey, H. Flyvbjerg, Optimal estimation of diffusion coefficients from single-particle trajectories. *Phys. Rev. E* **89**, 022726 (2014).

28. W. H. Otto, M. H. Keefe, K. E. Splan, J. T. Hupp, C. K. Larive, Analysis of molecular square size and purity via pulsed-field gradient NMR spectroscopy. *Inorg. Chem.* **41**, 6172–6174 (2002).

29. J. van Krugten, N. Danne, E. J. G. Peterman, A local interplay between diffusion and intraflagellar transport distributes TRPV-channel OCR-2 along *C. elegans* chemosensory cilia. *Commun. Biol.* **5**, 720 (2022).

30. Z. Zhang, N. Danne, B. Meddens, I. Heller, E. J. G. Peterman, Direct imaging of intraflagellar-transport turnarounds reveals that motors detach, diffuse, and reattach to opposite-direction trains. *Proc. Natl. Acad. Sci. U.S.A.* **118**, e2115089118 (2021).

31. M. Harterink, S. L. Edwards, B. de Haan, K. W. Yau, S. van den Heuvel, L. C. Kapitein, K. G. Miller, C. C. Hoogenraad, Local microtubule organization promotes cargo transport in *C. elegans* dendrites. *J. Cell Sci.* **131**, jcs223107 (2018).

32. H.-T. Chou, L. Apelt, D. P. Farrell, S. R. White, J. Woodsmith, V. Svetlov, J. S. Goldstein, A. R. Nager, Z. Li, J. Muller, H. Dollfus, E. Nudler, U. Stelzl, F. DiMaio, M. V. Nachury, T. Walz, The molecular architecture of native BBSome obtained by an integrated structural approach. *Structure* **27**, 1384–1394.e4 (2019).

33. S. Yang, K. Bahl, H.-T. Chou, J. Woodsmith, U. Stelzl, T. Walz, M. V. Nachury, Near-atomic structures of the BBSome reveal the basis for BBSome activation and binding to GPCR cargoes. *eLife* **9**, e55954 (2020).

34. H. Jin, S. R. White, T. Shida, S. Schulz, M. Aguiar, S. P. Gygi, J. F. Bazan, M. V. Nachury, The conserved Bardet-Biedl syndrome proteins assemble a coat that traffics membrane proteins to cilia. *Cell* **141**, 1208–1219 (2010).

35. F. Oswald, B. Prevo, S. Acar, E. J. G. Peterman, Interplay between ciliary ultrastructure and IFT-train dynamics revealed by single-molecule super-resolution Imaging. *Cell Rep.* **25**, 224–235 (2018).

36. M. V. Nachury, The molecular machines that traffic signaling receptors into and out of cilia. *Curr. Opin. Cell Biol.* **51**, 124–131 (2018).

37. C. L. Williams, J. C. McIntyre, S. R. Norris, P. M. Jenkins, L. Zhang, Q. Pei, K. Verhey, J. R. Martens, Direct evidence for BBSome-associated intraflagellar transport reveals distinct properties of native mammalian cilia. *Nat. Commun.* **5**, 5813 (2014).

38. S. C. Jana, S. Mendonca, P. Machado, S. Werner, J. Rocha, A. Pereira, H. Maiato, M. Bettencourt-Dias, Differential regulation of transition zone and centriole proteins contributes to ciliary base diversity. *Nat. Cell Biol.* **20**, 928–941 (2018).

39. M. Taschner, S. Bhogaraju, M. Vetter, M. Morawetz, E. Lorentzen, Biochemical mapping of interactions within the intraflagellar transport (IFT) B core complex: IFT52 binds directly to four other IFT-B subunits. *J. Biol. Chem.* **286**, 26344–26352 (2011).

40. M. Taschner, E. Lorentzen, The intraflagellar transport machinery. *Cold Spring Harb. Perspect. Biol.* **8**, a028092 (2016).

41. B. Lv, L. Wan, M. Taschner, X. Cheng, E. Lorentzen, K. Huang, Intraflagellar transport protein IFT52 recruits IFT46 to the basal body and flagella. *J. Cell Sci.* **130**, 1662–1674 (2017).

42. C. J. Wiens, Y. Tong, M. A. Esmail, E. Oh, J. M. Gerdes, J. Wang, W. Tempel, J. B. Rattner, N. Katsanis, H.-W. Park, M. R. Leroux, Bardet-Biedl syndrome-associated small GTPase ARL6 (BBS3) functions at or near the ciliary gate and modulates Wnt signaling. *J. Biol. Chem.* **285**, 16218–16230 (2010).

43. X. Liu, Q. Shen, T. Yu, H. Huang, Z. Zhang, J. Ding, Y. Tang, N. Xu, S. Yue, Small GTPase Arl6 controls RH30 rhabdomyosarcoma cell growth through ciliogenesis and Hedgehog signaling. *Cell Biosci.* **6**, 61 (2016).

44. B. Xue, Y.-X. Liu, B. Dong, J. L. Wingfield, M. Wu, J. Sun, K. F. Lechtreck, Z.-C. Fan, Intraflagellar transport protein RABL5/IFT22 recruits the BBSome to the basal body through the GTPase ARL6/BBS3. *Proc. Natl. Acad. Sci. U.S.A.* **117**, 2496–2505 (2020).

45. F. Ye, A. R. Nager, M. V. Nachury, BBSome trains remove activated GPCRs from cilia by enabling passage through the transition zone. *J. Cell Biol.* **217**, 1847–1868 (2018).

46. Y.-X. Liu, B. Xue, W.-Y. Sun, J. L. Wingfield, J. Sun, M. Wu, K. F. Lechtreck, Z. Wu, Z.-C. Fan, Bardet-Biedl syndrome 3 protein promotes ciliary exit of the signaling protein phospholipase D via the BBSome. *eLife* **10**, e59119 (2021).

47. A. Olivier-Mason, M. Wojtyniak, R. V. Bowie, I. V. Nechipurenko, O. E. Blacque, P. Sengupta, Transmembrane protein OSTA-1 shapes sensory cilia morphology via regulation of intracellular membrane trafficking in *C. elegans*. *Development* **140**, 1560–1572 (2013).

48. M. Fujiwara, T. Teramoto, T. Ishihara, Y. Ohshima, S. L. McIntire, A novel zf-MYND protein, CHB-3, mediates guanylyl cyclase localization to sensory cilia and controls body size of *Caenorhabditis elegans*. *PLOS Genet.* **6**, e1001211 (2010).

49. R. O'Hagan, M. Silva, K. C. Q. Nguyen, W. Zhang, S. Bellotti, Y. H. Ramadan, D. H. Hall, M. M. Barr, Glutamylation regulates transport, specializes function, and sculpts the structure of cilia. *Curr. Biol.* **27**, 3430–3441.e6 (2017).

50. O. I. Kaplan, D. B. Doroquez, S. Cevik, R. V. Bowie, L. Clarke, A. A. W. M. Sanders, K. Kida, J. Z. Rappoport, P. Sengupta, O. E. Blacque, Endocytosis genes facilitate protein and membrane transport in *C. elegans* sensory cilia. *Curr. Biol.* **22**, 451–460 (2012).

51. D. B. Doroquez, C. Berciu, J. R. Anderson, P. Sengupta, D. Nicastro, A high-resolution morphological and ultrastructural map of anterior sensory cilia and glia in *Caenorhabditis elegans*. *eLife* **3**, e01948 (2014).

52. O. E. Blacque, A. A. W. M. Sanders, Compartments within a compartment: What *C. elegans* can tell us about ciliary subdomain composition, biogenesis, function, and disease. *Organogenesis* **10**, 126–137 (2014).

53. Y.-C. Hsiao, K. Tuz, R. J. Ferland, Trafficking in and to the primary cilium. *Cilia* **1**, 4 (2012).

54. Z. Anvarian, K. Mykytyn, S. Mukhopadhyay, L. B. Pedersen, S. T. Christensen, Cellular signalling by primary cilia in development, organ function and disease. *Nat. Rev. Nephrol.* **15**, 199–219 (2019).

55. D. Serwas, T. Y. Su, M. Roessler, S. Wang, A. Dammermann, Centrioles initiate cilia assembly but are dispensable for maturation and maintenance in *C. elegans*. *J. Cell Biol.* **216**, 1659–1671 (2017).

56. J. M. Brown, D. A. Cochran, B. Craige, T. Kubo, G. B. Witman, Assembly of IFT trains at the ciliary base depends on IFT74. *Curr. Biol.* **25**, 1583–1593 (2015).

57. S. Meleppattu, H. Zhou, J. Dai, M. Gui, A. Brown, Mechanism of IFT-A polymerization into trains for ciliary transport. *Cell* **185**, 4986–4998.E12 (2022).

58. S. E. Lacey, H. E. Foster, G. Pigino, The molecular structure of IFT-A and IFT-B in anterograde intraflagellar transport trains. *Nat. Struct. Mol. Biol.* **30**, 584–593 (2023).

59. S. J. Hesketh, A. G. Mukhopadhyay, D. Nakamura, K. Toropova, A. J. Roberts, IFT-A structure reveals carriages for membrane protein transport into cilia. *Cell* **185**, 4971–4985.e16 (2022).

60. W. Fu, L. Wang, S. Kim, J. Li, B. D. Dynlacht, Role for the IFT-A complex in selective transport to the primary cilium. *Cell Rep.* **17**, 1505–1517 (2016).

61. T. Hirano, Y. Katoh, K. Nakayama, Intraflagellar transport-A complex mediates ciliary entry and retrograde trafficking of ciliary G protein–coupled receptors. *Mol. Biol. Cell* **28**, 429–439 (2017).

62. T. Picariello, J. M. Brown, Y. Hou, G. Swank, D. A. Cochran, O. D. King, K. Lechtreck, G. J. Pazour, G. B. Witman, A global analysis of IFT-A function reveals specialization for transport of membrane-associated proteins into cilia. *J. Cell Sci.* **132**, jcs220749 (2019).

63. P. Liu, K. F. Lechtreck, The Bardet-Biedl syndrome protein complex is an adapter expanding the cargo range of intraflagellar transport trains for ciliary export. *Proc. Natl. Acad. Sci. U.S.A.* **115**, E934–E943 (2018).

64. W. Mul, A. Mitra, B. Prevo, E. J. G. Peterman, DYF-5 regulates intraflagellar transport by affecting train turnaround. bioRxiv 612404 [Preprint] (2024). https://doi.org/10.1101/2024.09.11.612404.

65. J. Burghoorn, M. P. J. Dekkers, S. Rademakers, T. de Jong, R. Willemsen, G. Jansen, Mutation of the MAP kinase DYF-5 affects docking and undocking of kinesin-2 motors and reduces their speed in the cilia of *Caenorhabditis elegans*. *Proc. Natl. Acad. Sci. U.S.A.* **104**, 7157–7162 (2007).

66. C. Frokjaer-Jensen, M. W. Davis, C. E. Hopkins, B. J. Newman, J. M. Thummel, S.-P. Olesen, M. Grunnet, E. M. Jorgensen, Single-copy insertion of transgenes in *Caenorhabditis elegans*. *Nat. Genet.* **40**, 1375–1383 (2008).

67. A. Paix, A. Folkmann, G. Seydoux, Precision genome editing using CRISPR-Cas9 and linear repair templates in *C. elegans*. *Methods* **121–122**, 86–93 (2017).

68. S. Brenner, The genetics of *Caenorhabditis elegans*. *Genetics* **77**, 71–94 (1974).

69. E. Loseva, J. van Krugten, A. Mitra, E. J. G. Peterman, Single-molecule fluorescence microscopy in sensory cilia of living *Caenorhabditis elegans*. *Methods Mol. Biol.* **2694**, 133–150 (2024).

70. H. Deschout, K. Neyts, K. Braeckmans, The influence of movement on the localization precision of sub-resolution particles in fluorescence microscopy. *J. Biophotonics* **5**, 97–109 (2012).

71. F. Ruhnow, D. Zwicker, S. Diez, Tracking single particles and elongated filaments with nanometer precision. *Biophys. J.* **100**, 2820–2828 (2011).

72. N. Danné, Z. Zhang, E. J. G. Peterman, Classifying directed and diffusive transport in short, noisy single-molecule trajectories with wMSD. bioRxiv 513659 [Preprint] (2022). https://doi.org/10.1101/2022.10.25.513659.

73. J. Mijalkovic, B. Prevo, F. Oswald, P. Mangeol, E. J. G. Peterman, Ensemble and single-molecule dynamics of IFT dynein in *Caenorhabditis elegans* cilia. *Nat. Commun.* **8**, 14591 (2017).
